# Modeling and simulation of optical wireless communication channels in IoUT considering water types turbulence and transmitter selection

**DOI:** 10.1038/s41598-025-10935-w

**Published:** 2025-08-04

**Authors:** M. Mokhtar Zayed, Mona Shokair

**Affiliations:** 1 Department of Communications, Faculty of Electronic Engineering, Menoufia University, Menoufia Governorate, Menouf City, Egypt; 2 Department of Communications and Computers Engineering, Higher Institute of Engineering, El-Shorouk Academy, El-Shorouk City, Egypt; 3 Department of Electrical Engineering, Faculty of Engineering, October 6 University, Giza Governorate, October 6 City, Egypt

**Keywords:** Internet of underwater things (IoUT), Optical wireless communication (OWC), Data transmission, Communication optimization, Channel modeling, Turbulence effects, Underwater environments, Engineering, Optics and photonics, Physics

## Abstract

The Internet of Underwater Things (IoUT) is revolutionizing underwater communication by enabling real-time data exchange, environmental monitoring, and exploration in aquatic environments. Among emerging technologies, optical wireless communication (OWC) has gained prominence due to its high-speed data rates and superior efficiency compared to traditional acoustic and radio frequency (RF) methods. This paper presents a comprehensive study of OWC channel modeling and simulation tailored for IoUT applications. The research investigates the physical characteristics of underwater optical channels, focusing on the effects of absorption, scattering, turbulence, and various noise sources on light propagation across diverse water types, including pure seawater, clear coastal waters, and turbid harbor waters. A central aspect of the study is the comparative evaluation of two transmitter types—light-emitting diode photo sources (LED-PS) and laser diode photo sources (LD-PS)—both operating at a 520 nm wavelength (green light). Their performance is assessed under varying environmental conditions, incorporating three turbulence models: log-normal, generalized gamma, and Weibull distributions. Simulation models are developed and implemented using MATLAB and Python to analyze key parameters such as transmission distance, water type, transmitter characteristics, wavelength, and turbulence intensity. Performance metrics, including received optical power, signal-to-noise ratio (SNR), and bit error rate (BER), are evaluated to provide in-depth insights into system behavior. Results show that LD-PS consistently outperforms LED-PS across all scenarios. For instance, at a received power threshold of − 53.4 dBm, LD-PS achieves a communication distance of up to 68.39 m in pure seawater (compared to 27.36 m for LED-PS), while in turbid harbor, the range is reduced to 3.08 m. At a BER of 10^−5^, LD-PS reaches 67.69 m in pure seawater and 3.18 m in turbid harbor conditions. Under a fixed SNR of 50 dB, LD-PS achieves a maximum range of 73.34 m in pure sea. The minimum SNR required to maintain a BER of 10^−5^ is 12.19 dB in pure seawater and rises to 91.94 dB in turbid harbor conditions. These findings advance the development of OWC systems by providing practical guidelines for optimizing underwater communication performance. The insights presented serve as a foundation for designing robust and efficient IoUT networks capable of reliable data transmission across a range of aquatic environments.

## Introduction

IoUT is transforming underwater communication by enabling smart, interconnected networks of devices designed to operate in aquatic environments. These systems are pivotal for a wide range of applications, including environmental monitoring, underwater exploration, disaster prevention, military operations, and navigation assistance^1–3^. However, one of the biggest challenges in IoUT deployment is establishing reliable, high-speed communication links, as traditional methods—RF and acoustic communication—face inherent limitations. Acoustic communication provides long-range coverage but suffers from low data rates, high latency, and severe signal attenuation^4,5^. RF communication, on the other hand, is heavily attenuated in water—especially in conductive saltwater—rendering it impractical for long-distance transmission^6,7^. In contrast, OWC has emerged as a compelling alternative, offering high data rates, low latency, and minimal interference over short to medium distances^8,9^. By utilizing visible light and infrared wavelengths, OWC systems can achieve significantly higher bandwidth than RF-based methods^10,11^. Despite these advantages, underwater OWC performance is highly dependent on environmental factors, including water type, absorption and scattering effects, turbulence-induced fluctuations, and noise sources such as background shot noise and thermal noise^12,13^. These complexities highlight the need for robust modeling and simulation frameworks to accurately predict and optimize OWC system performance in diverse underwater conditions.

The growing demand for high-speed, reliable underwater communication has intensified interest in OWC as a core enabler for IoUT applications. However, many existing studies focus either on simplified theoretical models or limited experimental setups, often overlooking the dynamic and heterogeneous nature of underwater optical channels. The diverse optical properties of different water types, combined with turbulence and noise effects, make accurate performance prediction a significant challenge. To address this gap, this paper proposes a holistic and parameter-sensitive simulation framework for UOWC system evaluation. Unlike prior works that examine isolated channel effects or specific source types, our model simultaneously incorporates real-world water conditions, statistical turbulence behavior, noise characteristics, and transmitter-specific beam properties. This comprehensive approach enables more realistic performance assessment and supports the refinement of OWC system design and deployment strategies. Ultimately, the proposed framework contributes to the development of more robust and efficient IoUT networks capable of reliable data transmission across a wide range of underwater environments.

This paper makes the following significant contributions:

1. *Comprehensive OWC channel modeling*:

- Develops an advanced modeling framework integrating key underwater optical factors, including absorption, scattering, turbulence effects, and noise sources.
- Provides a detailed performance evaluation across multiple water types and environmental conditions.

2. *Extensive simulation of performance metrics*:

- Conducts in-depth simulations to assess key OWC performance indicators, such as BER, SNR, and received optical power.
- Explores variations in water conditions, optical source characteristics, and system configurations, offering practical design insights.

3. *Application to IoUT systems*:

- Presents actionable guidelines for optimizing OWC-based IoUT networks, ensuring efficient and reliable underwater communication.
- Demonstrates applicability in real-world scenarios, including environmental monitoring, disaster prevention, and underwater exploration.

4. *Comparison with traditional communication methods*:

- Provides a comparative analysis between OWC, acoustic, and RF communication technologies.
- Highlights OWC’s superior performance in terms of data rate, power efficiency, operational range, and reliability for underwater applications (see Table 1).

**Table 1:** Comparison of underwater wireless communication technologies^14,15,42^.

Feature	RF communication	Acoustic communication	Optical communication
Propagation Speed	2.3 × 10^8^ m/s (Near light speed)	1500 m/s (Speed of sound in water)	2.3 × 10^8^ m/s (Near light speed)
Data Transmission Rate	Up to Mbps	Limited to Kbps	Up to Gbps
Available Bandwidth	MHz range	0.001–0.1 MHz	Up to 150 MHz
Communication Latency	Moderate	High (Significant delay)	Low (Near-instantaneous)
Operating Frequency	30–300 MHz	0.01–0.015 MHz	5 × 10^8^ MHz (Visible/Infrared spectrum)
Effective Range	≤ 10 m	Can exceed 100 km	10–150 m (Dependent on water clarity)
Transmitter Power	Milliwatts to Watts	Typically, ~ 10 W	Milliwatts to Watts
Signal Attenuation	Depends on frequency & conductivity (3.5–5 dB/m)	Varies with distance & frequency (0.1–4 dB/km)	0.39 dB/m (clear ocean), 11 dB/m (turbid water)
Advantages	- Effective for near-surface applications. - Moderate data rates achievable at short distances. - Less affected by water turbulence.	- Most widely used for long-range underwater communication. - Effective over distances up to 20 km or more.	- Ultra-high-speed data transfer (Gbps). - Minimal latency, ideal for real-time applications. - Compact, low-cost transceivers.
Challenges	- Severely range-limited in conductive seawater. - High energy consumption. - Large, bulky transceivers.	- Extremely low data rates (Kbps). - High signal delay (seconds per transmission). - Large, energy-intensive equipment. - Potential impact on marine life.	- Highly susceptible to absorption & scattering. - Limited range (10–150 m, depending on water conditions). - Cannot penetrate the air-water interface efficiently.

Considering the insights from Table 1, optical communication emerges as the preferred choice for underwater applications due to its exceptional data transmission speeds (up to Gbps), nearly negligible latency, and cost-effective, compact transceiver design. These characteristics make it ideal for high-speed data transfer within moderate-range underwater networks. However, optical communication faces challenges such as limited propagation distance, high absorption, scattering effects, and difficulty in traversing the water-air interface. To overcome these limitations, system optimization strategies must be employed, including wavelength selection tailored to specific water conditions, advanced beam-forming techniques, and the integration of hybrid communication architectures that combine optical links with RF or acoustic methods to enhance range and reliability.

This study provides a comprehensive examination of underwater optical wireless communication (UOWC) channel modeling and simulation, emphasizing the impact of environmental factors on system performance. By addressing these challenges, this research contributes to the advancement of next-generation IoUT networks, ensuring more efficient and robust underwater communication systems for diverse applications, including environmental monitoring, underwater exploration, and disaster response.

This study uniquely contributes a multi-dimensional performance framework that simultaneously considers multiple real-world water types, statistical turbulence models, and photo-source variations in a unified simulation environment. Unlike previous studies, which focused on either simplified water conditions or a single turbulence model, our work enables cross-comparative analysis that reflects the physical realities of diverse underwater environments. This approach allows us to define meaningful performance thresholds for UOWC system design.

The rest of the paper is structured as follows: Sect. 2 presents a comprehensive literature review, discussing existing studies on UOWC and highlighting advancements in system modeling, modulation techniques, and performance enhancement methods. Section 3 explores water types, detailing their optical properties such as absorption and scattering coefficients, which significantly impact signal propagation. Section 4 examines various IoUT applications, demonstrating the relevance of UOWC in environmental monitoring, underwater exploration, and military operations. Section 5 provides a block diagram of the UOWC system, illustrating the fundamental components and their interactions. Section 6 elaborates on the architecture of the UOWC system, describing the structural design and operational workflow. Section 7 introduces the proposed system model, outlining key innovations and methodologies employed to enhance communication efficiency. Section 8 delves into aquatic channel modeling, discussing mathematical formulations for signal propagation, noise factors, and performance constraints in different water conditions. Section 9 presents simulation results and discussions, analyzing key performance metrics such as BER, SNR, and received power under varying conditions. Finally, Sect. 10 concludes the study, summarizing the key findings and proposing directions for future work to further improve UOWC system performance through advanced modulation schemes and AI-based optimization techniques.

## Literature reviews

OWC has emerged as a promising solution for high-speed data transmission in the IoUT, offering advantages over traditional acoustic and radio frequency methods. However, the performance of underwater OWC is significantly influenced by factors such as water type, turbulence effects, and transmitter selection. Existing studies have explored various modeling approaches to characterize attenuation, scattering, and noise sources in different underwater environments. This section reviews key advancements in underwater OWC channel modeling and simulation, highlighting the impact of environmental conditions and system configurations on communication performance.

The study in^16^ modeled the UOWC channel by simultaneously considering absorption, scattering, and turbulence effects using Monte Carlo simulation and phase screen approaches. Results showed that increasing seawater turbidity and turbulence intensity caused greater signal dispersion, with turbulence introducing an additional 5 dB path loss. The channel impulse response (CIR) analysis revealed a 50% reduction in peak amplitude and significant temporal spreading, highlighting the critical impact of turbulence on UOWC system performance.

In^17^, the study evaluated OWC performance in air and water, analyzing received optical power experimentally and mathematically. Results showed greater attenuation in water than in air, with further degradation in UOWC performance due to air bubbles. Received optical power decreased with increasing channel length, and mathematical modeling aligned well with experimental findings, validating the optical channel analysis.

The authors of^18^ developed a comprehensive UOWC model considering turbulence, absorption, scattering, and various noise sources. Using Monte Carlo simulations, it analyzed BER and eye diagrams for clear, coastal, and harbor waters over an 8 m transmission span. Results showed that link performance degraded with increasing distance and water turbidity, with BER remaining near zero for clear and coastal waters but reaching 4.1 × 10^-3^ in harbor waters. Acceptable BER was maintained up to 30 m in clear, 15 m in coastal, and 6 m in harbor waters.

According to^19^, the study analyzed underwater optical communication using free-space optical wireless formulas, considering the optical path, refractive index, and water types. Results showed that received signal power, link margin, data rate, and SNR decreased as the refractive index and distance increased. Pure and clean water provided the highest signal power, link margin, and SNR, while turbid waters exhibited the most signal degradation.

Ref^20^. analyzed the transmission range limits of underwater visible light communication (UVLC) by developing a closed-form path loss expression based on water type, beam divergence, and receiver aperture. The model, validated through Monte Carlo simulations, showed that in non-turbid waters, UVLC can achieve distances up to 43.95 m, while increasing turbidity significantly reduces the range.

As noted in^21^, the study analyzed turbulence-induced fading in UOWC by evaluating various statistical distributions, including lognormal, Gamma, K, Weibull, and exponentiated Weibull. Using a Monte Carlo-based model, closed-form expressions for average BER and outage probability were derived. Results showed that as turbulence strength increased, differences between fading models became more pronounced, highlighting the need for accurate channel modeling in UOWC system design.

The authors of^22^ analyzed the performance of UOWC links by modeling weak turbulence with a log-normal distribution and moderate-to-strong turbulence with a gamma-gamma distribution. The Rytov variance and scintillation index were derived, and closed-form BER expressions for IM/DD with OOK modulation were obtained. Results showed that turbulence effects became comparable to absorption and scattering as intensity increased, significantly degrading performance. Various environmental and system parameters were examined, highlighting the need for mitigation techniques like adaptive optics and spatial diversity to maintain acceptable BER over tens of meters.

In^23^, the study modeled vertical UVLC links as cascaded fading channels due to ocean stratification, where non-mixing water layers have varying turbulence strengths. Using lognormal and gamma-gamma distributions, closed-form BER and ergodic capacity expressions were derived. Results showed that assuming constant turbulence strength leads to inaccurate BER estimations. The analysis revealed that diversity gain in gamma-gamma channels is dominated by the weakest layer, while all layers contribute in lognormal channels. The findings highlight the impact of layering on system performance and capacity.

The authors of^24^ investigated UOWC under different water quality conditions by developing a channel model and analyzing its impact on communication range. The model was validated through experiments, and sources of error were identified. The concept of “effective communication space” was introduced to assess spatial coverage beyond linear distance. Tests on an autonomous underwater vehicle (AUV) and field experiments in a lake confirmed the model’s applicability, providing insights for future engineering applications and advancements in UOWC technology.

As reported in^25^, the study proposed a composite channel model incorporating multi-size bubbles, absorption, and multiple scattering effects to analyze UOWC. Using Mie theory and Monte Carlo simulations, the model demonstrated that increased bubble density led to greater attenuation, lower received power, and changes in scattering properties. Comparisons with conventional particle scattering validated the model’s accuracy, providing valuable insights for designing more reliable UOWC links.

In^26^, the study modeled an underwater optical wireless communication channel using vector radiative transfer theory, incorporating multiple scattering and polarization effects. Monte Carlo simulations quantified inter-symbol interference and signal attenuation due to scattering and absorption. Results showed that increasing distance degraded BER performance, with multi-level amplitude modulation further worsening BER compared to on-off keying. These findings help predict feasible transmission distances based on bit rate and field of view.

According to^27^, the study integrated turbulence, absorption, and scattering effects into a unified Monte Carlo simulation framework for underwater optical wireless channels using the generalized Snell’s Law and a multiple phase screen model. The received intensity followed a lognormal distribution under weak turbulence, and power loss analysis highlighted the combined impact of these effects. The findings provided valuable insights for characterizing underwater optical communication.

The authors of^28^ developed a comprehensive multiparameter model for UOWC channels by integrating absorption, scattering, and turbulence effects using the Beer-Lambert law and Monte Carlo phase screens. The model achieved 96.19% accuracy in line with theoretical statistics. Results showed that BER performance could be improved by increasing transmission power under weak to medium turbulence, but this method became ineffective under strong turbulence. A power penalty of 5.8 dBm was observed over 50 m from pure seawater to ocean water and 1.0 dBm from weak to medium turbulence at a BER threshold of 10^-6^.

Ref^29^. developed a realistic UOWC channel model using Monte Carlo simulations to analyze impulse response and time dispersion under various water types and system parameters. Results showed that channel delay dispersion was negligible in most practical cases, even over 50 m in clear water, making the channel effectively frequency non-selective. Consequently, inter-symbol interference (ISI) was minimal, eliminating the need for complex signal processing like channel equalization at the receiver.

As noted in^30^, the study proposed a unified statistical model using the Exponential-Generalized Gamma (EGG) distribution to characterize turbulence-induced fading in UOWC channels due to air bubbles and temperature gradients in fresh and salty waters. The model, validated with experimental data, provided a perfect fit under all conditions and enabled closed-form expressions for key performance metrics such as outage probability, BER, and ergodic capacity. Results showed that increasing temperature gradients and air bubbles degraded UOWC system performance, highlighting the need for robust communication designs to enhance reliability and speed in underwater wireless networks.

In^31^, the study analyzed the effects of absorption, scattering, and turbulence in UOWC links by proposing an integrated stochastic model for the spatial distribution of optical intensity. The photodiode receiving process was modeled, and BER performance was evaluated. Results showed that stronger turbulence degrades BER, while MIMO can mitigate fading effects. Additionally, turbulence had a greater impact on BER than link geometry, and increasing transmitted power reduced MIMO’s diversity gain.

The study in^32^ investigated a 450 nm blue laser-based UOWC system, achieving a 1.25 Gbps data rate over a 6 m distance with BER values below 10^−8^. Experiments analyzed the impact of water turbulence, temperature variations, and artificial seawater. Results showed minimal BER impact from turbulence, optimal performance at 25 °C, and a reduced 3 m transmission range in artificial seawater due to increased scattering. These findings provide insights into UOWC performance under practical environmental conditions.

As noted in^33^, the study used Monte Carlo simulations to model absorption, scattering, and turbulence (AST) in underwater optical wireless communication. Results showed that optical turbulence followed a lognormal distribution under weak conditions but deviated with increasing turbulence. The impulse response (IR) remained unchanged when turbulence was ignored, while tripling the coastal link length from 10 to 30 m increased IR variance by over five times.

The study in^34^ modeled the multi-layer vertical UOWC link as a cascaded channel using various turbulence models (GG, EGG, EW, ΓΓ) and analyzed system performance through outage probability, average BER, and ergodic capacity. Asymptotic expressions determined system diversity order, while fixed-gain AF relaying integrated terrestrial OWC with UOWC. Monte Carlo simulations validated results, showing a performance gap between single-layer and multi-layer models, highlighting the impact of oceanic conditions on UOWC performance.

The authors of^35^ focused on analyzing the performance of a UVWOC link using OOK modulation under weak turbulence, pointing errors, and attenuation. The underwater channel was modeled with non-identical HTLN-distributed turbulent layers and Rayleigh-distributed pointing errors. Closed-form expressions for average BER, outage probability (OP), and ergodic channel capacity (ECC) were derived for both SISO and receive diversity schemes. Results showed that the MRC scheme offered the best BER performance, and increasing the number of detectors reduced transmit power requirements. Analytical results closely matched Monte Carlo simulations, validating the model’s accuracy.

The study in^36^ focused on modeling and analyzing UOWC channel performance under various underwater conditions using OOK modulation. It evaluated the effects of water type, absorption, scattering, turbulence (modeled with multiple statistical distributions), and diverse noise sources. Using a 450 nm LD-PS and an avalanche photodetector, key metrics like BER and received power were assessed. Results showed significant performance degradation over longer distances and in turbid waters, emphasizing the need for optimized system design to ensure reliable underwater optical communication.

In^37^, the study focused on analyzing and comparing various modulation techniques for UVLC systems under challenges like absorption, scattering, and turbulence. It evaluated SCM and MCM schemes, including OOK, PPM, QAM-CAP, OFDM, and space-domain index modulation, through simulations and experiments. Results showed that advanced modulation strategies significantly improved data rates (up to 30 Gbps over 21 m) and BER performance. The study highlighted the importance of modulation selection for spectral efficiency and system robustness while identifying key challenges in channel estimation and system optimization for dynamic underwater environments. Table 2 presents a comparative summary of the key aspects of the referenced studies.

**Table 2:** A succinct overview of the essential aspects of the reviewed studies.

Ref.	Study focus	Key methodology	Main findings	Key contributions
^16^	UOWC channel modeling considering absorption, scattering, and turbulence	Monte Carlo simulation, phase screen approach	Turbulence introduces 5 dB additional path loss; CIR shows 50% peak amplitude reduction	Highlights the critical impact of turbulence on signal dispersion
^17^	OWC in air and water	Experimental and mathematical analysis	Greater attenuation in water; air bubbles further degrade UOWC performance	Validates optical channel analysis via experimental results
^18^	BER and eye diagram analysis in UOWC for different water types	Monte Carlo simulation	BER remains near zero for clear/coastal waters; 4.1 × 10^-3^ in harbor water	Defines acceptable BER limits for different environments
^19^	Optical path and refractive index effects in UOWC	Free-space optical wireless formulas	Higher SNR and signal power in pure/clean water; degraded performance in turbid waters	Emphasizes water type’s role in UOWC link margin and data rate
^20^	UVLC transmission range limits	Closed-form path loss expression, Monte Carlo simulation	Max range of 43.95 m in non-turbid waters; increased turbidity reduces range	Develops a validated path loss model
^21^	Turbulence-induced fading in UOWC	Monte Carlo-based modeling	Strong turbulence amplifies fading model differences	Highlights the need for accurate turbulence modeling
^22^	Performance analysis of UOWC under turbulence	Log-normal and Gamma-Gamma distributions	Increased turbulence equates to absorption/scattering in performance degradation	Suggests mitigation techniques like adaptive optics
^23^	Vertical UVLC links in stratified waters	Cascaded fading channel modeling	Layered turbulence affects BER; weakest layer dominates in GG channels	Highlights layered turbulence impact on UOWC
^24^	UOWC under different water conditions	Experimental validation with AUV	Introduces “effective communication space” concept	Provides insights for real-world UOWC applications
^25^	Impact of multi-size bubbles on UOWC	Mie theory, Monte Carlo simulations	Increased bubble density lowers received power	Enhances accuracy in underwater bubble modeling
^26^	Vector radiative transfer theory in UOWC	Monte Carlo simulations	Scattering/absorption degrade BER; OOK outperforms multi-level modulation	Models inter-symbol interference in UOWC
^27^	Unified Monte Carlo-based UOWC simulation	Generalized Snell’s Law, phase screen model	Lognormal intensity distribution under weak turbulence	Characterizes power loss from multiple effects
^28^	Multi-parameter UOWC channel model	Beer-Lambert law, Monte Carlo phase screens	96.19% accuracy; 5.8 dBm power penalty over 50 m	Demonstrates power constraints in turbulence
^29^	UOWC channel impulse response and time dispersion	Monte Carlo simulations	Negligible ISI over 50 m in clear water	Minimizes need for channel equalization
^30^	Exponential-Generalized Gamma (EGG) model for turbulence	Experimental validation	Temperature gradients and bubbles degrade performance	Proposes a unified fading model
^31^	Integrated stochastic modeling of UOWC	BER and photodiode analysis	Strong turbulence degrades BER; MIMO mitigates fading	Highlights impact of MIMO on turbulence
^32^	Blue laser-based UOWC system	Experimental analysis	Achieves 1.25 Gbps over 6 m with BER < 10^-8^	Shows practical environmental impacts on UOWC
^33^	AST modeling in UOWC	Monte Carlo simulations	Lognormal turbulence holds for weak conditions	Quantifies impulse response variations
^34^	Multi-layer vertical UOWC link	Various turbulence models (GG, EGG, EW, ΓΓ)	Multi-layer models differ significantly from single-layer	Demonstrates diversity order in vertical UOWC links
^35^	UVWOC link under turbulence, pointing errors, and attenuation	HTLN turbulence model, Rayleigh pointing errors, closed-form BER/OP/ECC for SISO & diversity schemes	MRC offered best BER; more detectors reduced power need; results matched simulations	Derived accurate BER, OP, ECC expressions; validated via Monte-Carlo simulations
^36^	UOWC performance under environmental conditions	Statistical turbulence models (Log-Normal, Gamma, etc.), noise modeling, LD-PS & APD setup	Performance degraded in turbid/long-distance links; reliable comms needs optimized design	Provided in-depth channel modeling and BER/power analysis under realistic underwater conditions
^37^	Modulation strategies for UVLC	Comparison of SCM/MCM (OOK, PPM, QAM-CAP, OFDM); simulations & experiments	Advanced schemes (e.g., OFDM) achieved up to 30 Gbps over 21 m; modulation choice critical for robustness	Identified optimal modulations; emphasized challenges in channel estimation and system optimization

## Water types

**Fig. 1:**
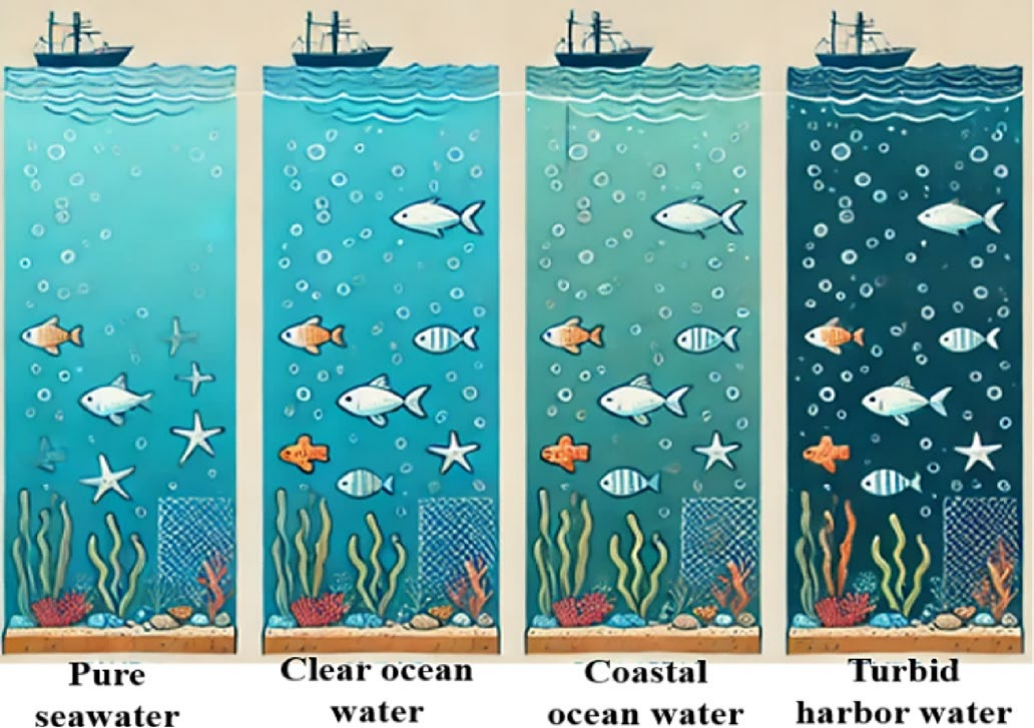
Various water types.

Water types in UOWC significantly impact signal propagation due to varying levels of absorption and scattering. *Pure seawater* has the lowest attenuation, allowing for the longest transmission distances with minimal signal degradation. *Clear ocean water* exhibits slightly higher absorption and scattering than pure seawater but still supports relatively long-range communication. *Coastal ocean water* contains more suspended particles and organic matter, leading to increased scattering and reduced optical transmission efficiency. *Turbid harbor water* experiences the highest level of attenuation due to a high concentration of sediments, pollutants, and biological matter, severely limiting optical communication range and requiring advanced mitigation techniques^36,38^. Figure 1 presents a diagram depicting various water types, transitioning from pure seawater to turbid harbor water.

## IoUT applications

**Fig. 2:**
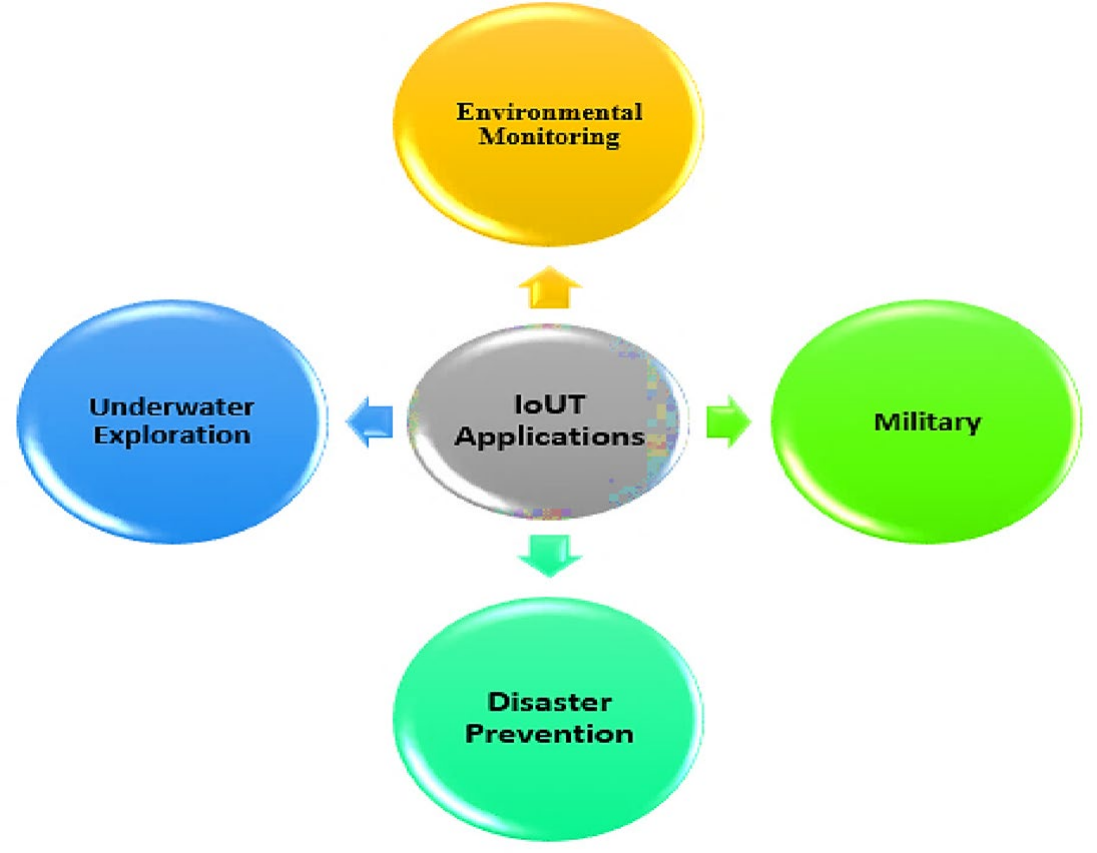
Various IoUT applications.

IoUT is revolutionizing oceanic research and industrial operations by enabling seamless communication between underwater devices, sensors, and autonomous systems. IoUT plays a crucial role in environmental monitoring, allowing scientists to track ocean temperatures, salinity, pollution levels, and marine biodiversity in real time. It is also widely applied in underwater exploration, supporting deep-sea research, offshore resource mapping, and archaeological discoveries. In disaster prevention, IoUT aids in early detection of tsunamis, underwater earthquakes, and structural failures in offshore platforms. Military and defense sectors utilize IoUT for surveillance, submarine communication, and mine detection. Additionally, IoUT enhances navigation assistance for underwater vehicles and supports maritime industries by enabling real-time tracking and automated inspections of underwater infrastructure. These applications demonstrate the growing significance of IoUT in advancing underwater technology and sustainable ocean management^39,40^. Figure 2 showcases a diagram illustrating the diverse applications of IoUT across multiple domains.

## Block diagram of UOWC system

**Fig. 3:**
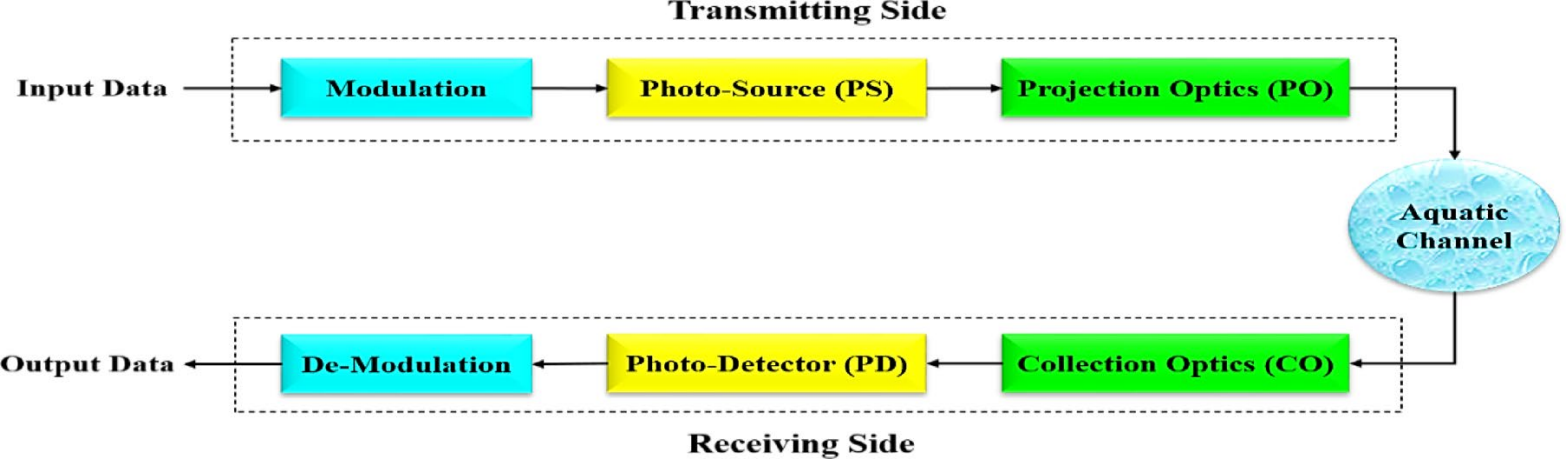
The block diagram of the basic components of UOWC system.

In the UOWC system, the process begins with input data, which represents the digital information that needs to be transmitted. This data undergoes modulation, where it is encoded using modulation schemes such as On-Off Keying (OOK), Quadrature Amplitude Modulation (QAM), or Phase Shift Keying (PSK) to adapt it for optical transmission. The modulated signal is then fed into the photo source (PS), which consists of an optical transmitter, typically an LED or LD, that emits the modulated optical signal. To ensure efficient propagation through the underwater environment, the emitted light passes through projection optics (PO), which shape and direct the optical beam for optimal transmission. The aquatic channel serves as the medium through which the optical signal travels. However, this channel introduces impairments such as absorption, scattering, and turbulence, which can degrade signal quality and limit communication range. At the receiver end, Collection Optics (CO), consisting of optical lenses or concentrators, capture and focus the transmitted light onto the photodetector. The photodetector, which can be an avalanche photodiode (APD) or a PIN photodiode, converts the received optical signal into an electrical signal. This electrical signal then undergoes demodulation, where the original data is recovered by decoding the modulated information. Finally, the processed signal is output as received data, completing the transmission process. Figure 3 illustrates the block diagram of the fundamental components of a UOWC system.

## Architecture of UOWC system

**Fig. 4:**
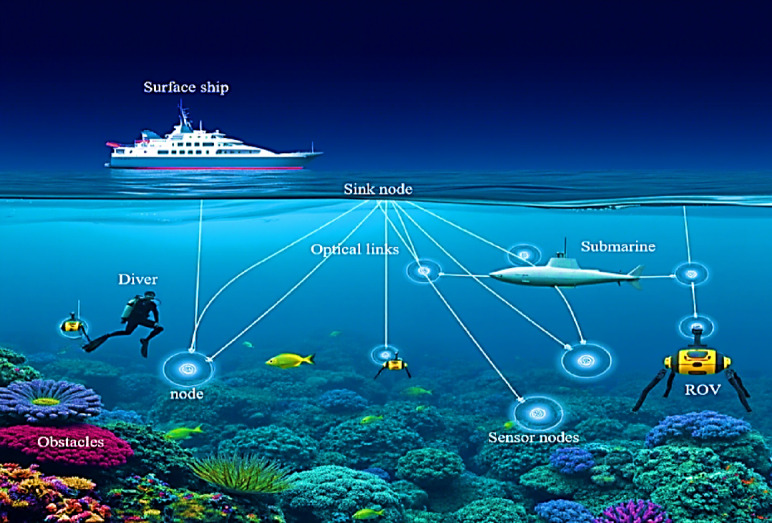
Architecture of UOWC system.

A typical UOWC system comprises a surface node, which can be a ship, buoy, or a surface autonomous vehicle (SAV), and an underwater node located at various depths. The underwater node may consist of a submarine, autonomous underwater vehicle (AUV), unmanned underwater vehicle (UUV), diver, or a network of sensors deployed on the seabed. These nodes communicate using optical wireless links, allowing high-speed data transmission. The collected data is relayed from the underwater node to the surface node, which then forwards it to a remote monitoring station for further processing and analysis. Figure 4 illustrates the architecture of a standard UOWC system.

## The proposed system model

**Fig. 5:**
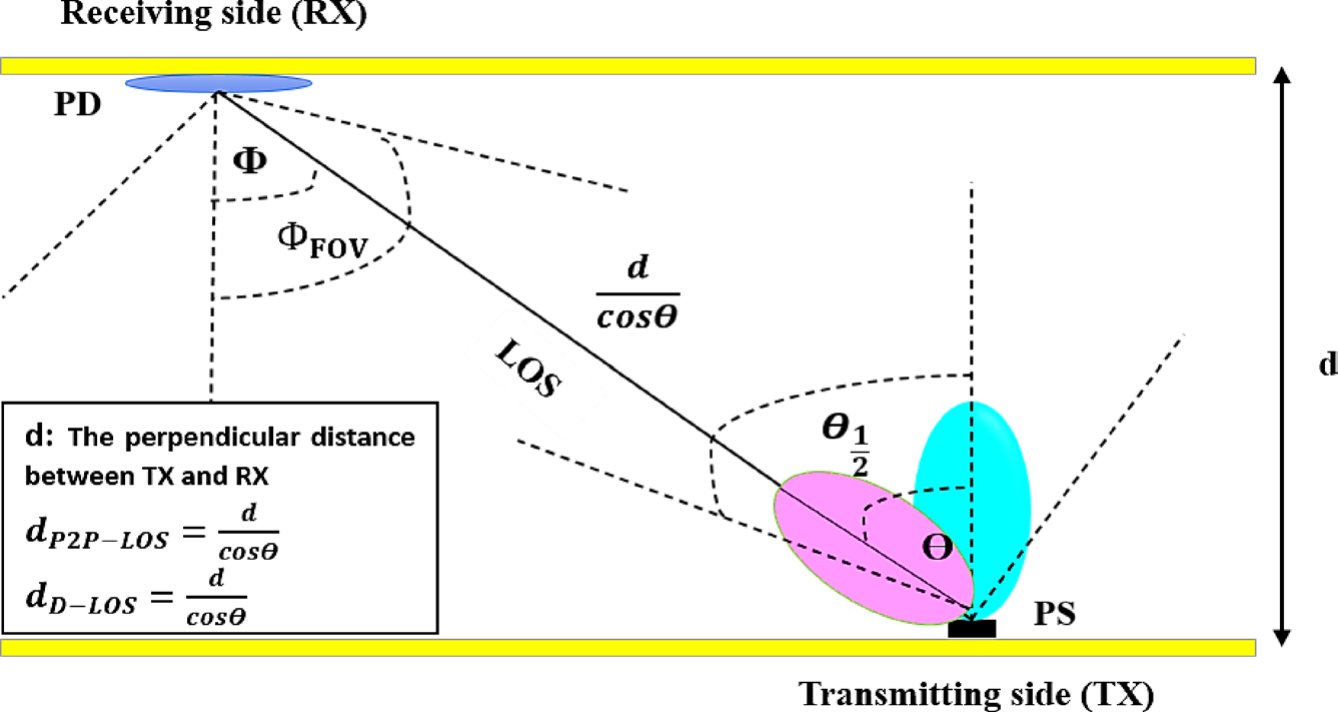
The proposed system model for different angles.

This study explores underwater VLC in uplink scenarios, where data transmission occurs from the ocean floor to the surface. As depicted in Fig. 5, the system consists of a source node positioned on the seabed, emitting an optical signal with a beam divergence angle (*θ*) and a semi-angle at half power (*θ*_*1/2*_). The transmitted light propagates through the water column and reaches the sink node at the surface, where it is influenced by the incident angle (*ϕ*), the receiver’s field of view (FOV) angle (*ϕ*_*FOV*_), and the effective communication range (*d*). This study employs a deterministic, physics-based simulation approach using defined system parameters and environmental models to evaluate UOWC performance.

**Fig. 6:**
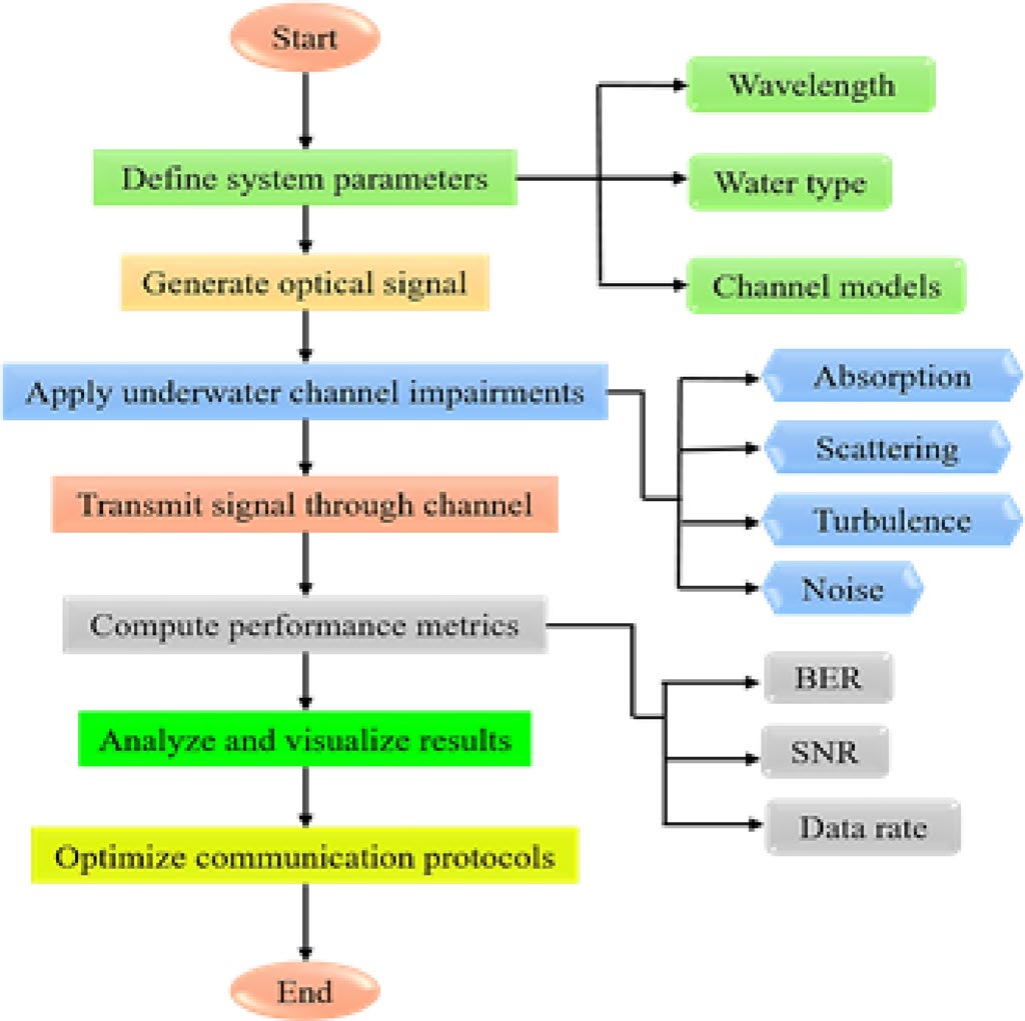
The proposed system’s flowchart.

The flowchart presented in this paper follows a structured approach, beginning with the definition of system parameters and environmental conditions, including wavelength, water type, and channel models. Subsequently, the optical signal is generated and subjected to underwater channel impairments such as absorption, scattering, and turbulence. The signal is then transmitted through the channel, after which key performance metrics—including SNR, BER, and data rate—are computed. Finally, the results are analyzed and visualized to evaluate system performance and refine communication protocols for IoUT applications. This methodology ensures a thorough assessment of the optical communication system’s efficiency in underwater environments. The proposed system’s flowchart is depicted in Figure 6.

## Aquatic channel modeling

**Fig. 7:**
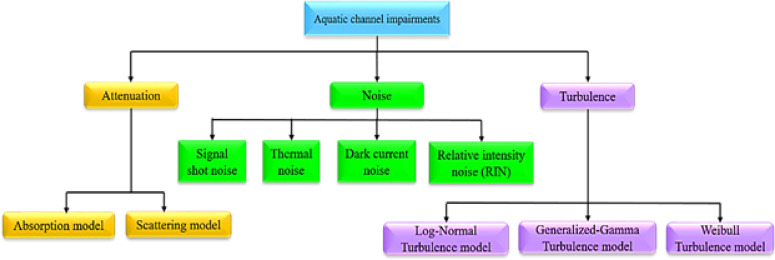
Aquatic channel impairments.

An aquatic channel refers to the medium through which signals propagate in underwater communication systems. Unlike terrestrial wireless channels, aquatic channels exhibit unique and complex characteristics due to the presence of water molecules, suspended particles, and varying environmental conditions. Signal transmission in such channels is significantly affected by absorption, scattering, turbulence, and bio-optical properties of the water, which vary depending on factors such as depth, temperature, salinity, and water type (e.g., clear ocean, coastal, or turbid waters). These impairments result in signal attenuation, reduced SNR, and increased BER, posing challenges for reliable data transmission. Moreover, the choice of communication modality—whether acoustic, RF, or optical—depends on the operational requirements and environmental constraints. OWC is particularly advantageous in underwater environments due to its high bandwidth and low latency; however, it is highly susceptible to beam divergence, multiple scattering effects, and limited transmission range. Addressing these challenges requires advanced channel modeling, adaptive modulation schemes, and robust signal processing techniques to optimize data transmission efficiency for applications such as the IoUT, environmental monitoring, and underwater exploration. Figure 7 illustrates the various impairments affecting the aquatic channel in UOWCs.

### Absorption model

Absorption in an underwater environment is mainly caused by water molecules and other impurities, which convert optical energy into heat. The power loss due to absorption is modeled using Beer’s Law:

1 $$\:{I}_{abs}\left(d,\lambda\:\right)={I}_{0}\left(\lambda\:\right).$$

where $\:{I}_{abs}(d,\lambda\:)$ is the received intensity after distance *d* for wavelength *λ*, $\:{I}_{0}\left(\lambda\:\right)$ is the initial transmitted intensity, $\:\alpha\:\left(\lambda\:\right)$ is the absorption coefficient of water (m^−1^), and *d* is the propagation distance (m). The total absorption coefficient $\:\alpha\:\left(\lambda\:\right)$ is determined based on the type of water (pure, coastal, or turbid) and is obtained experimentally or from tabulated data.

### Scattering model

Scattering in UOWCs is caused by suspended particles and molecules that redirect the transmitted optical signal. The single scattering model follows:

2 $$\:{I}_{scat}\left(d,\lambda\:\right)={I}_{0}\left(\lambda\:\right){e}^{-\beta\:\left(\lambda\:\right)d}.$$

where $\:{I}_{scat}(d,\lambda\:$) is the received power considering scattering, $\:\beta\:\left(\lambda\:\right)$ is the scattering coefficient (m^−1^). The total extinction coefficient is the sum of absorption and scattering:

3 $$\:c\left(\lambda\:\right)=\alpha\:\left(\lambda\:\right)+\beta\:\left(\lambda\:\right).$$

Thus, the total attenuation of an optical signal due to absorption and scattering follows:

4 $$\:I\left(d,\lambda\:\right)={I}_{0}\left(\lambda\:\right){e}^{-c\left(\lambda\:\right)d}.$$

The scattering phase function *P(θ)* determines the angular redistribution of light:

5 $$\:P\left(\theta\:\right)=\frac{1-{g}^{2}}{{(1+{g}^{2}-2g\text{cos}\theta)}^{\frac{3}{2}}}.$$

where *θ* is the scattering angle, *g* is the anisotropy factor (0 for isotropic, near 1 for forward scattering). Table 3 presents the standard values of *α(λ)*, *β(λ)*, and *c(λ)* for different water types, specifically within the green spectrum at a wavelength of *λ* = 520 nm, considering both LED and LD photo-sources.

**Table 3:** Typical values of *α(λ)*,* β(λ)*, and *c(λ)* for different types of water at a wavelength of *λ* = 520 nm^14,40^.

Types of water	Absorption coefficient $\:\alpha\:\left(\lambda\:\right)\:\left({\text{m}}^{-1}\right)$	Scattering coefficient $\:\beta\:\left(\lambda\:\right)\:\left({\text{m}}^{-1}\right)$	Extinction coefficient $\:c\left(\lambda\:\right)\:\left({\text{m}}^{-1}\right)$
Pure Sea Water	0.04418	0.0009092	0.0450892
Clear Ocean Water	0.08642	0.01226	0.09868
Costal Ocean Water	0.2179	0.09966	0.31756
Turbid Harbor Water	1.112	0.5266	1.6386

**Fig. 8:**
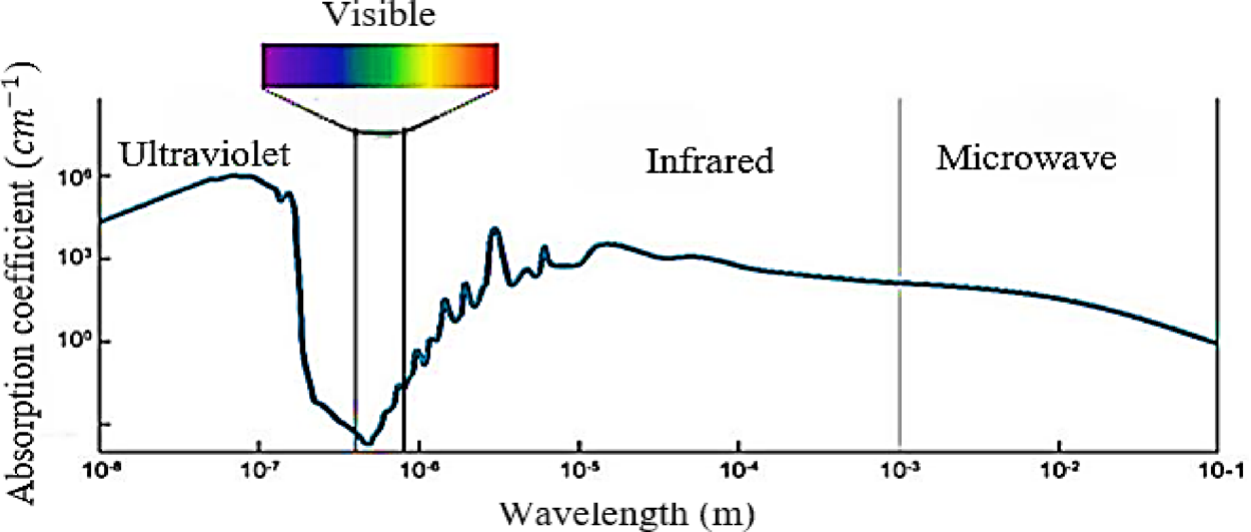
The light absorption coefficient in pure seawater varies as a function of wavelength^36,40^.

The blue-green region of the optical spectrum, particularly wavelengths between 450 nm (blue) and 520 nm (green), plays a crucial role in UOWC due to its minimal attenuation in seawater^8,9^. While blue wavelengths experience lower absorption in pure water, they are more susceptible to scattering, especially in coastal and turbid waters. Green wavelengths (around 520 nm) offer a balance between absorption and scattering, making them highly effective for UOWC. The benefits of green wavelengths include improved penetration depth, reduced signal degradation, and enhanced reliability in diverse underwater environments, making them ideal for long-range optical communication applications. Figure 8 depicts the fluctuation of the absorption coefficient of light in pure seawater across different wavelengths.

### Noise model

Noise in UOWCs consists of several independent components, including background shot noise, thermal noise, dark current noise, and relative intensity noise. The total noise power is given by:

6 $$\:{\sigma\:}_{total}^{2}={\sigma\:}_{shot}^{2}+{\sigma\:}_{thermal}^{2}+{\sigma\:}_{dark}^{2}+{\sigma\:}_{RIN}^{2}.$$

#### Background shot noise

Shot noise arises due to the quantum nature of light and is given by:

7 $$\:{\sigma\:}_{shot}^{2}=2qR{P}_{r}B.$$

where *q* is the electron charge (1.6 × 10^−19^ C), *R* is the photodetector responsivity (A/W), $\:{P}_{r}$ is the received optical power (W), and *B* is the electrical bandwidth (Hz).

#### Thermal noise

Thermal noise in the receiver circuit is given by:

8 $$\:{\sigma\:}_{thermal}^{2}=\frac{4kTB}{{R}_{L}}.$$

where *k* is the Boltzmann constant (1.38 × 10^−23^ J/K), *T* is the receiver temperature (K), and $\:{R}_{L}$ is the load resistance (Ω).

#### Dark current noise

Dark current noise, caused by unwanted currents in the photodetector, is modeled as:

9 $$\:{\sigma\:}_{dark}^{2}=2q{I}_{dark}B.$$

where $\:{I}_{dark}$ is the dark current (A).

#### Relative intensity noise (RIN)

For LD sources, relative intensity noise is given by:

10 $$\:{\sigma\:}_{RIN}^{2}=RIN\:{P}_{r}^{2}\:B.$$

where *RIN* is the relative intensity noise factor.

In the absence of empirical data, robustness in this simulation-based study is assessed by varying environmental and system conditions that directly affect noise levels. Specifically, we evaluate performance across different water types (pure sea to turbid harbor), each introducing progressively higher absorption and scattering coefficients, thereby increasing total noise variance. Furthermore, all noise components—including thermal, shot, dark, and RIN—are parameterized in the simulation, allowing us to observe their isolated and cumulative impact on signal degradation, SNR, and BER. This approach enables rigorous testing of system robustness under high-noise aquatic environments.

### Turbulence model

Turbulence in UOWCs is caused by variations in refractive index due to temperature, salinity, and pressure fluctuations. The irradiance fluctuation due to turbulence can be modeled using log-normal, generalized gamma, or Weibull distributions. The log-normal distribution is well-suited for modeling weak to moderate turbulence in UOWC systems^43,44^. In contrast, the generalized gamma distribution offers greater flexibility, making it adaptable to a wide range of turbulence conditions^22^. Meanwhile, the Weibull distribution is particularly useful for characterizing moderate to strong turbulence effects, capturing the intensity variations in more challenging underwater environments^30,45^.

The selection of log-normal, generalized gamma, and Weibull distributions is based on their proven suitability in modeling fading due to weak-to-moderate underwater turbulence. The generalized gamma model, in particular, offers flexible parameterization that can emulate other distributions. While models such as the K-distribution or Exponential-Gamma are effective in strong scattering regimes, our study focuses on practical UOWC scenarios with moderate turbulence over short to medium link distances (up to ~ 75 m). These three models strike a balance between analytical tractability and empirical relevance, as supported by^22,43–45^.

#### Log-normal model

The log-normal distribution models weak to moderate turbulence conditions in UOWC. The PDF is given by:

11 $$\:P\left(I\right)=\frac{1}{I\sqrt{2\pi\:{{\sigma\:}_{X}}^{2}}}\:exp\:\left(-\frac{{\left(Ln\:I-{\mu\:}_{x}\right)}^{2}}{2\:{{\sigma\:}_{X}}^{2}}\right)\:\:,\:\:\:\:\:\:\:I>0.$$

where *I* is the received optical intensity, $\:{\mu\:}_{x}$ and$\:\:{{\sigma\:}_{X}}^{2}$ are the mean and variance of the logarithmic intensity of (*I*).

12 $$\:{\mu\:}_{x}=Ln\:{I}_{o}-\frac{{{\sigma\:}_{X}}^{2}}{2}\:$$

13 $$\:{{\sigma\:}_{X}}^{2}=Ln\left(1+{{\sigma\:}_{I}}^{2}\right).$$

where $\:{I}_{o}$ is the mean received intensity, and $\:{{\sigma\:}_{X}}^{2}=Ln\left(1+{{\sigma\:}_{I}}^{2}\right)$ represents the variance of the log-amplitude fluctuation, where $\:{{\sigma\:}_{I}}^{2}$ is the scintillation index.

#### Generalized gamma model

The generalized gamma distribution is a flexible model that can represent a wide range of turbulence conditions. The PDF is:

14 $$\:P\left(I\right)=\frac{v}{{\alpha\:}^{\beta\:}\varGamma\:(\beta\:/v)\:}\:{I}^{\beta\:-1}\:exp\left(-{\left(\frac{I}{\alpha\:}\right)}^{v}\:\right)\:\:,\:\:\:\:\:I>0.$$

where *α* is the scale parameter, *β* is the shape parameter, *v* is an additional shaping factor that controls the tail behavior, and *Γ*(·) is the Gamma function. When *ν = 2*, the generalized gamma distribution reduces to the Nakagami-m distribution, and when *ν = 1*, it simplifies to the standard Gamma distribution.

#### Weibull model

The Weibull model is used to describe turbulence in various underwater environments, especially for moderate to strong fading. Its PDF is given by:

15 $$\:P\left(I\right)=\frac{k}{{\uplambda\:}}{\left(\frac{I}{{\uplambda\:}}\right)}^{k-1}\text{e}\text{x}\text{p}\left(-{\left(\frac{I}{{\uplambda\:}}\right)}^{k}\right)\:\:,\:\:\:\:\:\:I>0.$$

where $\:\lambda\:$ is the scale parameter and *k* is the shape parameter, which determines the fading severity. For k > 1, the distribution models less severe fading, while k < 1 represents stronger turbulence-induced fading effects.

### The received power

**Fig. 9:**
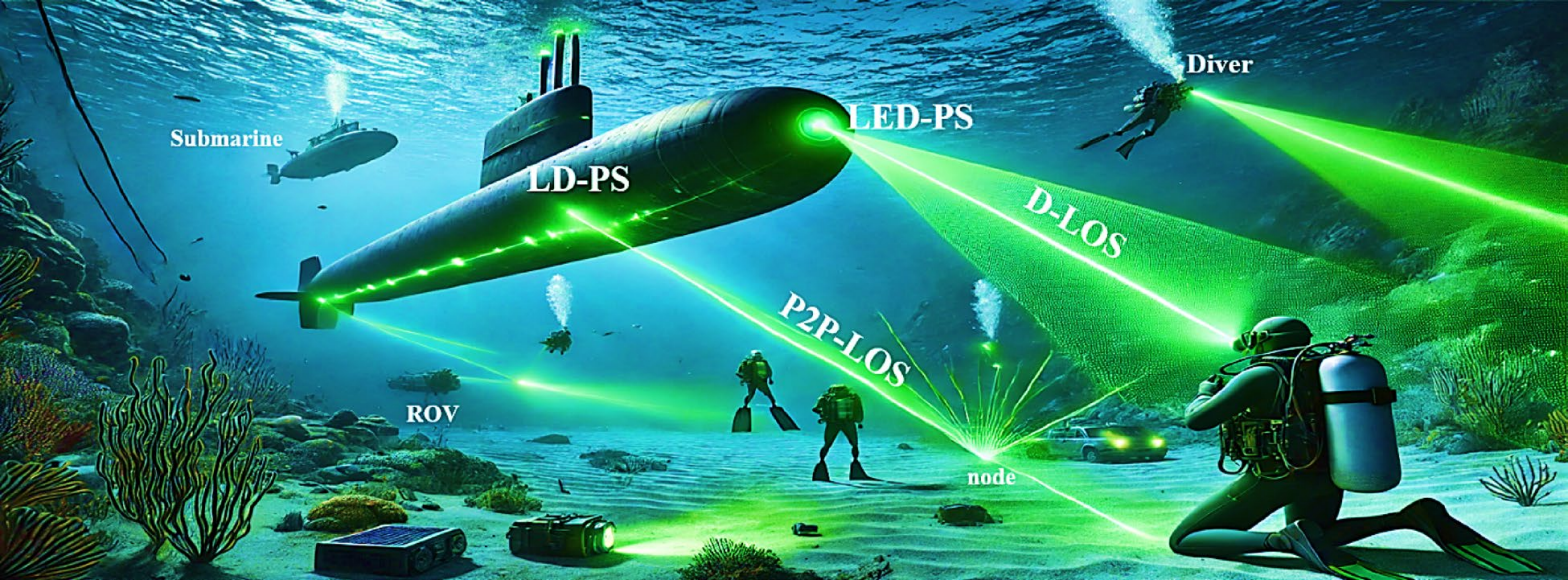
Point-to-point and Diffuse LoS link configuration in UOWC system.

In a point-to-point line-of-sight (LOS) scenario with an LD-PS, the received optical power is higher due to the highly directional beam, which minimizes scattering and maximizes power efficiency. This allows for long-range communication with low energy loss. However, LD-PS systems require precise alignment between the transmitter and receiver, making them susceptible to misalignment and beam wander due to underwater turbulence. In a diffuse LOS scenario with an LED-PS, the received optical power is lower due to wide beam divergence, leading to increased scattering and attenuation. While this reduces power efficiency and limits communication range, it provides better coverage and relaxed alignment requirements, making it more robust in dynamic underwater environments. Figure 9 visually illustrates the P2P-LOS and D-LOS link configurations in UOWC systems.

The LED-PS is modeled using a Lambertian emission pattern with wide beam divergence (~ 30°–120°), consistent with commercial high-brightness blue LEDs used in UOWC. In contrast, LD-PS is characterized by a narrower, quasi-Gaussian beam (~ 1°–10°), enabling higher directionality and greater reach. These source-specific properties influence received optical power and are supported by experimental findings in^46–48^. The simulation incorporates these angular and optical properties through source divergence parameters and geometric loss equations, ensuring accurate modeling of real-world underwater optical links.

#### Received power for LED-based photo source (LED-PS)

For an LED-based optical wireless communication system, the transmitted power exhibits a broader beam divergence. The received optical power at the photodetector is given by:

16 $$\:{P}_{r,\:\:\:LED-PS}={\:P}_{t\:}{\eta\:}_{t\:}{\eta\:}_{r}\:{e}^{-c\left(\lambda\:\right)d}\:{(\frac{(m+1)}{2\pi\:}{cos}}^{m}(\theta\:\left)\right){\:\:G}_{TXPO}\:{G}_{RXCO}\left(\phi\:\right)\:{T}_{S}\left(\phi\:\right)\:\:{F}_{turb\:\:\:}\frac{{A}_{PD}{cos}(\phi\:)}{2\pi\:{d}^{2}(1-cos \theta)}\:\varPi\:\left(\frac{\phi\:}{{\phi\:}_{FOV}}\right).$$

where

17 $$\:\varPi\:\left(\frac{\phi\:}{{\phi\:}_{FOV}}\right)=\left\{\begin{array}{c}\text{1}\text{}\text{}\text{}\text{}\text{}\text{}\text{}\text{}\text{}\text{}\text{}\text{}\text{}\text{}\text{}\frac{\phi\:}{{\phi\:}_{FOV}}\le\:1\\\:\text{}\text{}\text{}\text{}\text{0}\text{}\text{}\text{}\text{}\text{}\text{}\text{}\text{}\text{}\text{}\text{}\text{}\text{}\text{}\text{}\text{}\text{}\text{o}\text{t}\text{h}\text{e}\text{r}\text{w}\text{i}\text{s}\text{e}\text{}\text{}\text{}\end{array}\right.$$

Here, $\:{\:P}_{t\:}$represents the transmitted power, $\:{\eta\:}_{t\:}$and $\:{\eta\:}_{r}$​ are the transmitter and receiver efficiencies, respectively, and *c(λ)* is the total attenuation coefficient, accounting for both absorption and scattering losses. *θ* is the transmitter light beam divergence angle. The term $\:{\:\:G}_{TXPO}\:$​ denotes the gain of the transmitter projection optics, while $\:{G}_{RXCO}\left(\phi\:\right)$ represents the receiver collection optics gain. $\:{T}_{S}\left(\phi\:\right)$ is the transmission gain of the optical bandpass filter, and $\:{F}_{turb\:\:\:}$​ accounts for turbulence-induced fading. The term $\:{A}_{PD}$ refers to the active aperture area of the photodetector, and *d* is the link distance. The Lambertian order, *m*, which characterizes the LED radiation pattern, is given by:

18 $$\:m=\:\frac{-\text{l}\text{n}\left(2\right)}{\text{l}\text{n}\left(cos{\theta}_{\frac{1}{2}}\right)}.$$

where $\:{\theta}_{\frac{1}{2}}$​ represents the semi-angle at half power. The receiver collection optics gain is expressed as:

19 $$\:{G}_{RXCO}\left(\varPhi\:\right)=\left\{\begin{array}{cc}\frac{{n}^{2}}{{{sin}}^{2}{\varPhi\:}_{FOV}},&\:\text{0}\le\:\varPhi\:\le\:{\varPhi\:}_{FOV}\\\:\text{0}\text{,}&\:\varPhi\:>{\varPhi\:}_{FOV}\end{array}\right.$$

where *n* is the refractive index of the concentrator’s internal lens. A compound parabolic concentrator (CPC) with $\:{\varPhi\:}_{FOV}$ = π/6 is used in this study. For a Galilean beam expander, the transmitter optics gain is:

20 $$\:{G}_{TXPO}=\frac{f2}{\left|f1\right|}=\frac{{D}_{o}}{{D}_{i}}.$$

where *f1​* and *f2*​ are the focal lengths of the concave and convex lenses, respectively, and $\:{D}_{i}$​ and $\:{D}_{o}$​ represent the input and output beam diameters. Similarly, for a Keplerian beam expander, the gain is:

21 $$\:{G}_{TXPO}=\frac{f2}{f1}=\frac{{D}_{o}}{{D}_{i}}.$$

where *f1* is the focal length of the convex lens at the input. For simplicity, the optical narrow BPF transmission gain is assumed to be nearly constant and is set to $\:{T}_{S}\left(\phi\:\right)\:$= 1.

#### Received power for LD-based photo source (LD-PS)

For a LD-PS, the beam divergence is significantly lower than that of an LED. The received power is given by:

22 $$\:{P}_{r,\:\:\:LD-PS}={P}_{t\:}{\eta\:}_{t}{\:\eta\:}_{r}\:{e}^{-c\left(\lambda\:\right)d}\:{\:\:G}_{TXPO}\:{G}_{RXCO}\left(\phi\:\right)\:{T}_{S}\left(\phi\:\right){\:\:F}_{turb\:\:\:}\frac{{A}_{PD}{cos}(\phi\:)}{\pi\:{d}^{2}({tan}\theta\:{)}^{2}}\:\varPi\:\left(\frac{\phi\:}{{\phi\:}_{FOV}}\right)\text{}.$$

where all parameters retain their previously defined meanings, with the primary distinction being the focused nature of the laser beam, reflected in the denominator term $\:({tan}\theta\:{)}^{2}.$

### Signal-to-noise ratio (SNR) accounting for noise

To incorporate noise effects into the signal analysis, the SNR can be defined as:

23 $$\:SNR=\frac{{\left(R{P}_{r}\right)}^{2}}{{\sigma\:}_{total}^{2}}.$$

where *R* is the photodetector responsivity (A/W), $\:{P}_{r}$ is the received optical power (W), $\:{\sigma\:}_{total}^{2}$ is the total noise variance. The total noise variance consists of shot noise, thermal noise, dark current noise, and relative intensity noise (RIN).

### Evaluation of link performance

The performance of UOWC links is typically assessed using the BER, which is dependent on both SNR and turbulence-induced distortions. A widely used approximation for BER in optical communication systems in the case of OOK is given by:

24 $$\:BER=Q\left(\sqrt{\frac{{R}^{2}{{P}_{r}}^{2}}{{\sigma\:}_{total}^{2}}}\:\right)\simeq\:Q\left(\sqrt{SNR}\:\right).$$

where *Q(x)* is the Q-function and is given by:

25 $$\:Q\left(x\right)=\frac{1}{\sqrt{2\pi\:}}\:\:\underset{x}{\overset{\infty\:}{\int\:}}\text{e}\text{x}\text{p}(-\frac{{t}^{2}}{2})\:dt.$$

where *x* is a real-valued threshold or variable of interest. It is the lower limit of the integral and usually represents the SNR or a normalized decision variable in communication systems. *t* is a dummy variable of integration. It is used within the integral and has no meaning outside the integration bounds.

### Comprehensive channel model

A realistic UOWC channel must account for multiple attenuation mechanisms, including absorption, scattering, and turbulence. The overall channel response can be modeled as:

26 $$\:h\left(t\right)={h}_{abs}\:\:.\:\:{h}_{scat\:\:\:\:}.{\:h}_{turb}\left(t\right).$$

where $\:{h}_{abs}$ represents signal attenuation due to absorption, $\:{h}_{scat\:\:}$accounts for losses from scattering, and $\:{\:h}_{turb}\left(t\right)\:$models the dynamic variations induced by turbulence over time.

The received signal at any given time *t* can then be expressed as:

27 $$\:y\left(t\right)=h\left(t\right).x\left(t\right)+n\left(t\right).$$

where *x(t)* is the transmitted signal and *n(t)* represents the additive noise affecting the system.

Algorithm 1 represented a structured pseudocode that outlines the step-by-step execution of the model. This pseudocode clearly presents how input parameters are processed, how each impairment is applied to the signal model, and how performance outputs are computed and stored.

**Algorithm 1:**
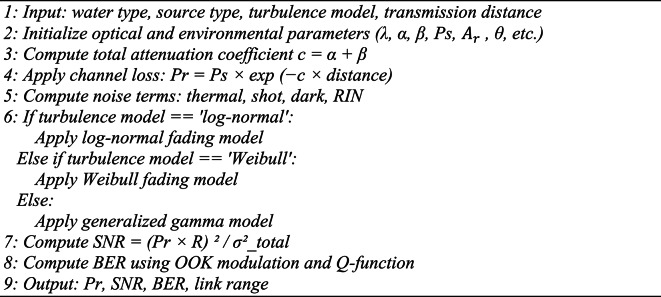
UOWC performance simulation.

## Simulation results and discussions

The performance evaluation in this study is based on parametric, deterministic simulations implemented in MATLAB and Python. All system and environmental parameters—such as water type, optical source characteristics, turbulence models, and channel configurations—are explicitly defined and systematically varied to assess their impact on key physical-layer metrics, including BER, SNR, and received optical power. The simulation framework was designed to operate efficiently on a standard computing setup and was capable of generating high-resolution performance plots across a wide range of underwater conditions. The main simulation parameters used in the analysis are summarized in Table 4.

**Table 4:** Simulation parameters.

Parameter	Symbol	Value	
Wavelength	$\:\lambda\:$	520 nm	
Photo source (PS)	*-*	LED-PS and LD-PS	
Color	*-*	Green light, optimal for underwater communication	
Transmitted power	$\:{P}_{t}$	1 W	
Transmitter efficiency	$\:{\eta\:}_{t}$	0.9	
Channel bandwidth	*B*	1 MHz	
Transmitter semi-angle at half power for D- LOS	$\:{\theta\:}_{\frac{1}{2}}$	60°	
Lambertian order for D-LOS	*m*	1	
Transmitter light beam divergence angle	*ϴ*	LED-PS	30°
		LD-PS	9°
Total attenuation coefficient	$\:c\left(\lambda\:\right)$	0.0450892, 0.09868, 0.31756, and 1.6386 m^−1^	
receiver efficiency	$\:{\:\eta\:}_{r}$	0.9	
Receiver light beam incident angle	*Ф*	LED-PS	15°
		LD-PS	5°
Receiver FOV angle [8, 9]	$\:{\varPhi\:}_{FOV}$	A hemispherical immersion lens	90°
		CPC	30°
Transmission distance	*d*	1 to 100 m	
Receiver Aperture Area	$\:{A}_{PD}$	1 mm^2^	
Refractive index of lens at PD	*n*	1.5	
Photo detector (PD)	*-*	SiPM-PD	
Receiver sensitivity^41^	$\:{P}_{s}$	-53.4 dBm	
Modulation scheme	*-*	On-off keying (OOK)	
Turbulence Models	-	Log-normal, Generalized-Gamma, Weibull distribution	
Noise sources	-	Thermal, Shot, Dark, and RIN	
Noise model	-	Additive white gaussian noise (AWGN)	

The transmit power was set to 1 W to reflect an upper-bound scenario aligned with previous experimental works^46,48^. However, we acknowledge that many IoUT applications operate with stricter energy constraints. For mobile or battery-powered nodes, practical transmit powers range from 100 to 300 mW. The impact of lower transmit power on received signal and BER is discussed in Sect. 9. Additionally, the use of a compound parabolic concentrator (CPC) with a fixed, narrow field-of-view (FOV) is modeled to reflect receiver designs optimized for directionality and high signal gain^8,9,46^. We recognize this setup may limit flexibility in dynamic node environments and have included this consideration in our system assumptions discussion. In addition, there are many studies^14,38^ that have examined the variation of the FOV angle and its impact on the link range and the received power.

**Fig. 10:**
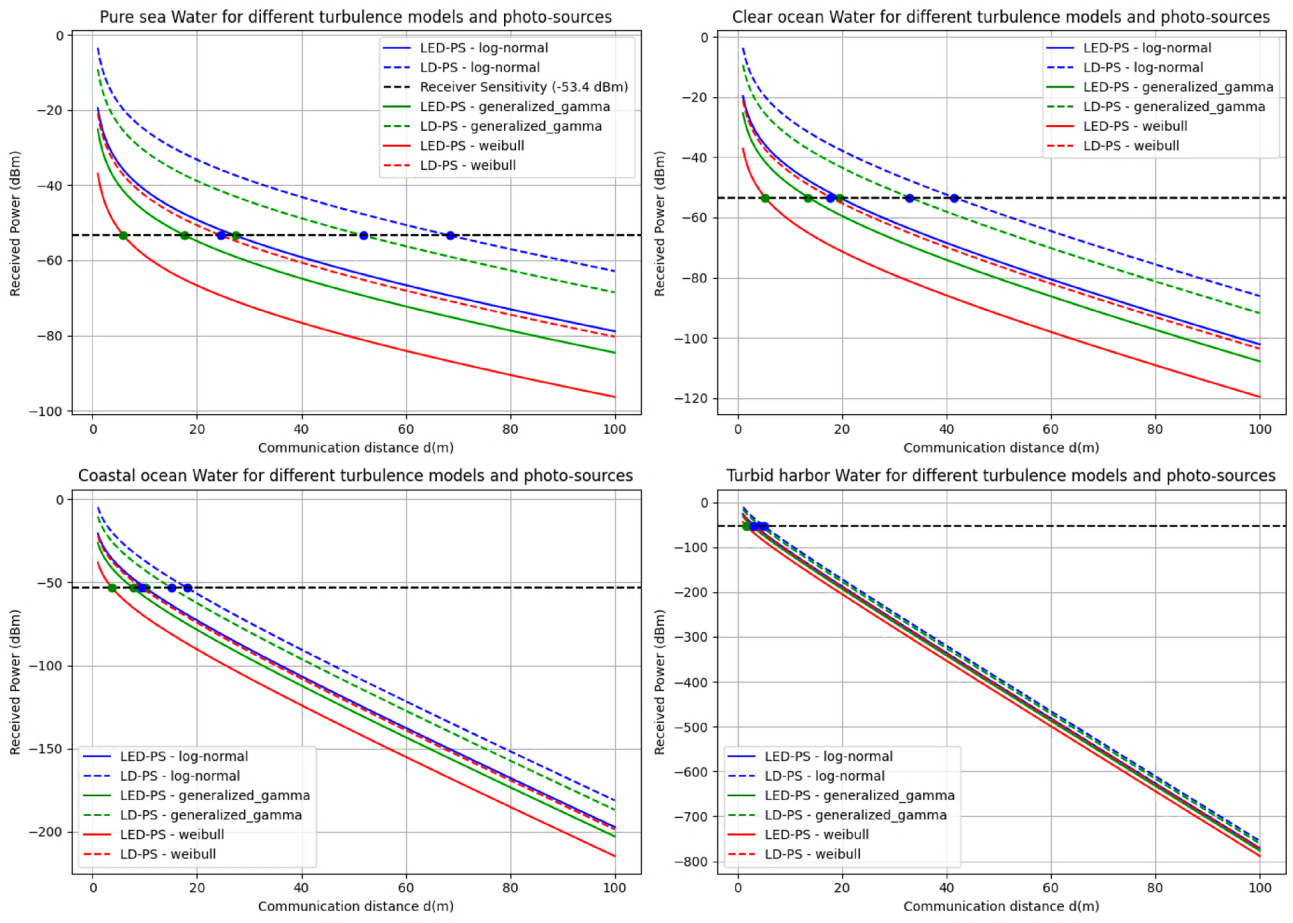
The received power (dBm) versus communication distance (m) for different water types under various turbulence models and photo-sources.

Figure 10 presents the received power (dBm) versus communication distance (m) for different water types (pure sea, clear ocean, coastal ocean, and turbid harbor) under various turbulence models (log-normal, generalized gamma, and Weibull) and photo-sources (LED-PS and LD-PS). It demonstrates that received power decreases with increasing distance, with higher losses in turbid harbor water. LD-PS generally performs better than LED-PS across all models. The receiver sensitivity threshold (-53.4 dBm) is marked, indicating the maximum achievable communication range for each scenario.

The results in Table 5 provide a comparative analysis of the achievable link range for LED-PS and LD-PS under different turbulence models across various water types at a receiver sensitivity of -53.4 dBm. The findings reveal significant variations in communication performance based on environmental conditions and turbulence characteristics.

According to the results in Table 5, the following can be concluded:

**Table 5:** The achievable link range for LED-PS and LD-PS under different turbulence models across various water types at ($\:{P}_{s}$ = -53.4 dBm)

Water type	Turbulence model	Link range for (LED-PS) in (m) at $\:{P}_{s}$ = -53.4 dBm	Link range for (LD-PS) in (m) at $\:{P}_{s}$ = -53.4 dBm
Pure Sea	Log-Normal	27.36	68.39
	Generalized Gamma	17.65	51.84
	Weibull	5.86	24.59
Clear Ocean	Log-Normal	19.43	41.43
	Generalized Gamma	13.49	32.91
	Weibull	5.16	17.75
Coastal Ocean	Log-Normal	10.12	18.05
	Generalized Gamma	7.74	15.07
	Weibull	3.68	9.42
Turbid Harbor	Log-Normal	3.28	5.06
	Generalized Gamma	2.68	4.37
	Weibull	1.69	3.08

### Impact of water type on link range

As water turbidity increases, the link range decreases. In pure seawater, which has the lowest absorption and scattering coefficients, the maximum link range is achieved. Turbid harbor water exhibits the most severe signal degradation, leading to reduced link ranges for both LED-PS and LD-PS. For instance, under log-normal turbulence, the link range in pure seawater is 27.36 m (LED-PS) and 68.39 m (LD-PS), while in turbid harbor water it drops to 3.28 m (LED-PS) and 5.06 m (LD-PS). This shows the adverse effect of high scattering and absorption in optically dense waters.

### Comparison of LED-PS and LD-PS performance

LD-PS consistently achieves a longer link range compared to LED-PS across all water types and turbulence models. This is due to its higher coherence, directional emission, and greater intensity, resulting in lower beam divergence and enhanced resistance to optical turbulence. For example, in clear ocean water under log-normal turbulence, the LD-PS link range (41.43 m) is more than twice that of LED-PS (19.43 m).

### Influence of turbulence models on link range

Among the three turbulence models, log-normal yields the longest link range, followed by generalized gamma, while Weibull results in the shortest. The reduced range in Weibull turbulence is due to its more severe fading characteristics. In turbid harbor water, the link range under Weibull turbulence drops to 1.69 m (LED-PS) and 3.08 m (LD-PS). This trend suggests better resilience under log-normal turbulence, especially in clear waters.

### Practical implications

The findings emphasize the importance of selecting appropriate sources and operating conditions based on the underwater environment. In clear waters, both LED-PS and LD-PS provide extended link ranges, making them suitable for medium-range communication. In turbid conditions, optical communication becomes limited, requiring strategies such as higher power, adaptive beamforming, or hybrid acoustic-optical solutions.

Overall, the study highlights the critical role of water turbidity and turbulence in determining the feasibility of underwater optical wireless communication. Future research should explore advanced modulation schemes and beamforming techniques to mitigate turbulence effects and enhance reliability.

**Fig. 11:**
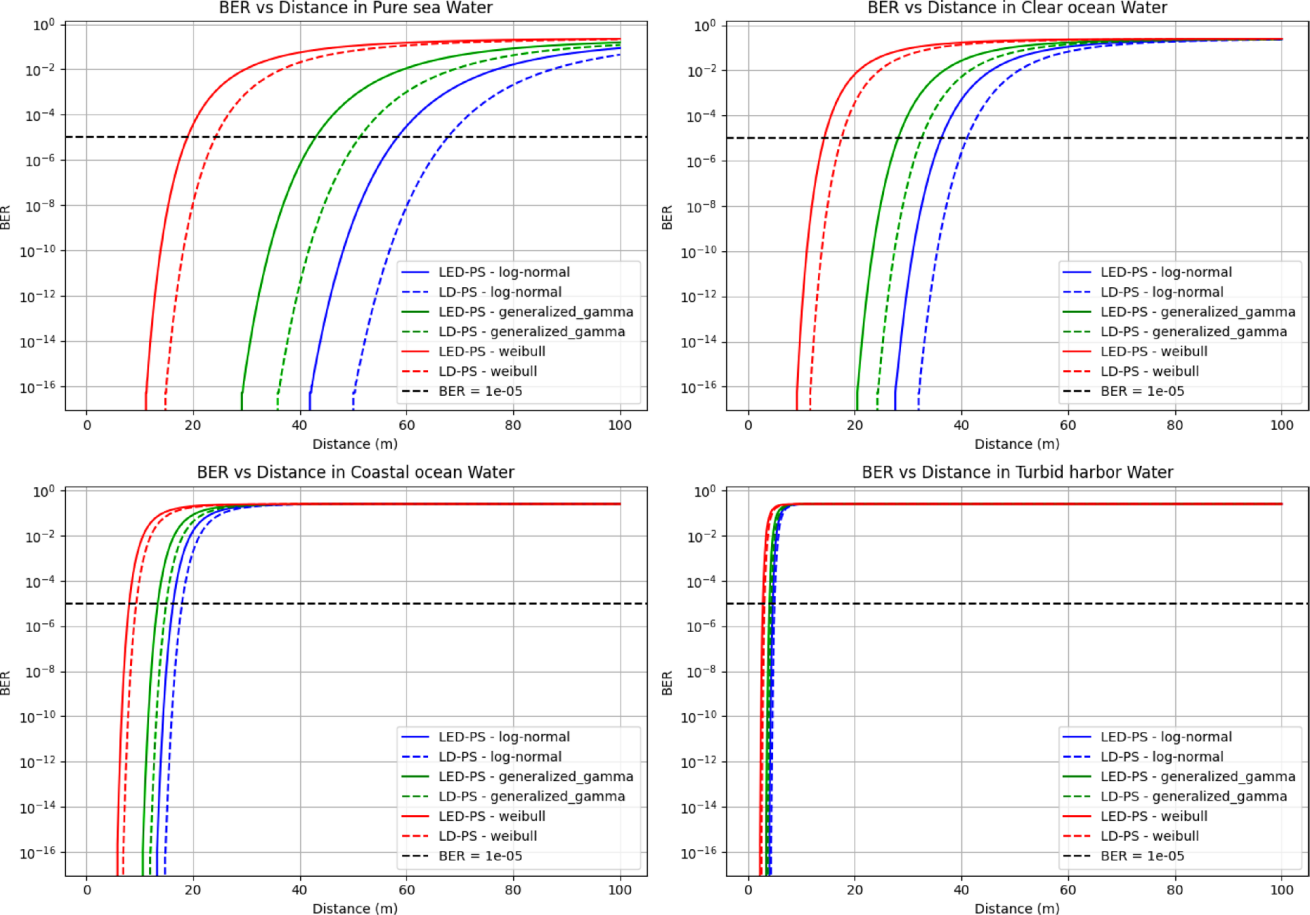
BER versus communication distance (m) for different water types under various turbulence models and photo-sources.

Figure 11 presents BER vs. distance for different water types under various turbulence models for LED-PS and LD-PS. BER increases with distance, with severe degradation in turbid waters. The threshold BER = 10^-5^ (black dashed line) marks the maximum transmission range for each scenario.

Table 6 presents the achievable link range for LED-PS and LD-PS under different turbulence models across various water types at a BER threshold of 10^-5^. The results show variations in link range based on water type, turbulence model, and source type.

**Table 6:** The achievable link range for LED-PS and LD-PS under different turbulence models across various water types at (BER = $\:{10}^{-5}$)

Water type	Turbulence model	Link range for (LED-PS) in (m) at BER = 10^−5^	Link range for (LD-PS) in (m) at BER = 10^−5^
Pure Sea	Log-Normal	58.28	67.69
	Generalized Gamma	42.92	51.24
	Weibull	18.94	24.19
Clear Ocean	Log-Normal	36.28	41.04
	Generalized Gamma	28.15	32.61
	Weibull	14.28	17.55
Coastal Ocean	Log-Normal	16.26	17.95
	Generalized Gamma	13.39	14.97
	Weibull	8.04	9.42
Turbid Harbor	Log-Normal	4.67	5.06
	Generalized Gamma	4.07	4.37
	Weibull	2.78	3.18

According to Table 6, the following can be concluded:

In *pure seawater*, with the lowest attenuation, the longest link range is achieved. The log-normal model yields the highest ranges, with LED-PS reaching 58.28 m and LD-PS 67.69 m. Generalized gamma shows a moderate reduction, while Weibull exhibits the shortest ranges, limiting LED-PS to 18.94 m and LD-PS to 24.19 m.

In *clear ocean water*, increased scattering and absorption reduce the range. The log-normal model maintains the longest distances (36.28 m for LED-PS, 41.04 m for LD-PS), followed by generalized gamma. Weibull shows a notable reduction (14.28 m LED-PS, 17.55 m LD-PS).

In *coastal ocean water*, a further reduction is seen. Log-normal supports 16.26 m (LED-PS) and 17.95 m (LD-PS), while Weibull reduces this to 8.04 m and 9.42 m, respectively.

In *turbid harbor water*, the most challenging environment, all models show severely limited ranges. Log-normal allows 4.67 m (LED-PS) and 5.06 m (LD-PS), while Weibull drops this to 2.78 m and 3.18 m.

LD-PS consistently outperforms LED-PS due to its higher power, lower divergence, and better collimation. Water type strongly influences range, with pure seawater allowing the longest distances and turbid harbor water the shortest. The turbulence model also significantly affects performance, with log-normal being the most favorable and Weibull the most restrictive. These results underscore the importance of source and turbulence model selection when designing UOWC systems.

**Fig. 12:**
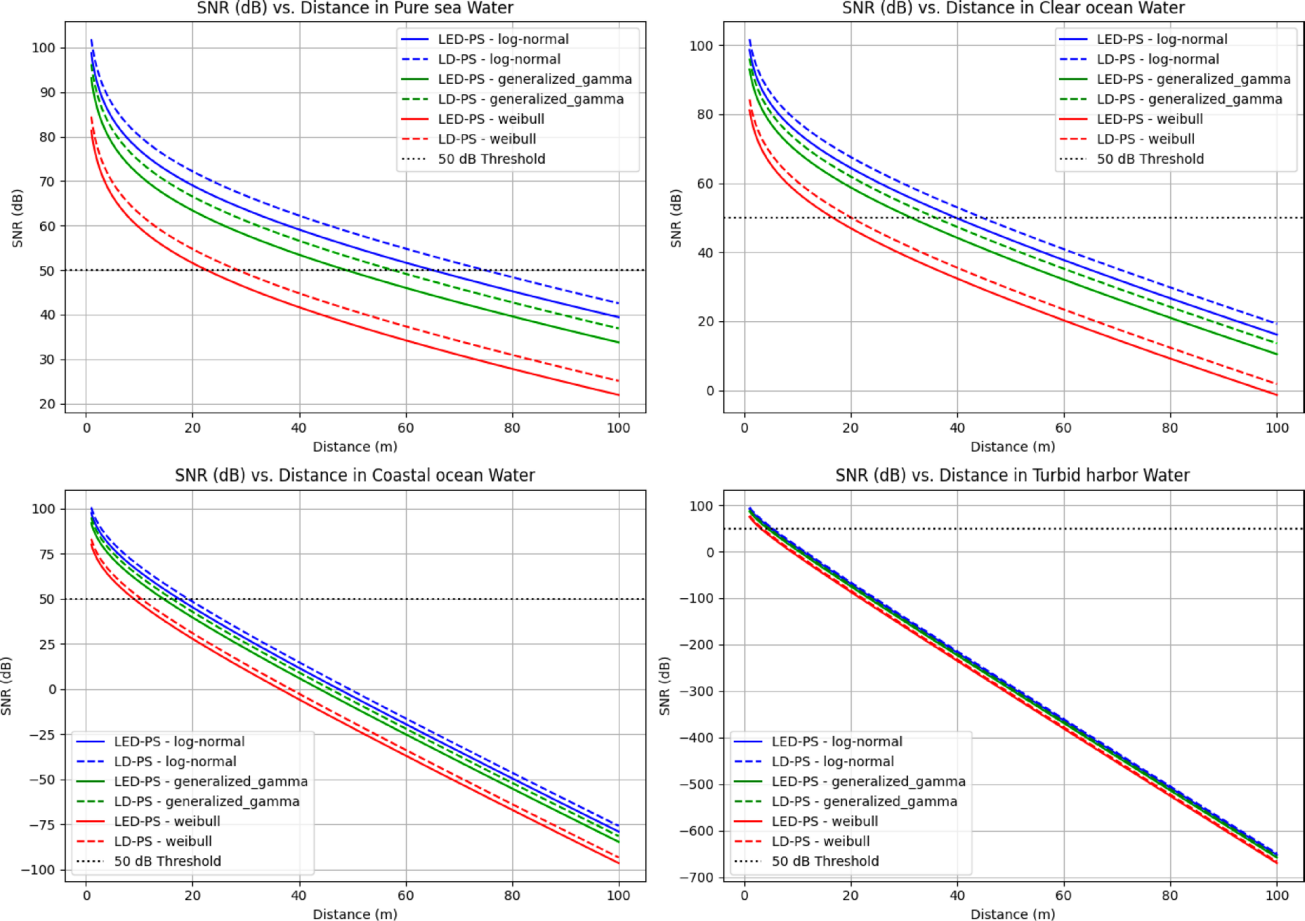
SNR (dB) versus communication distance (m) for different water types under various turbulence models and photo-sources.

Figure 12 shows SNR (dB) versus distance (m) for different turbulence models in four water types using LED-PS and LD-PS. SNR decreases with distance, especially in turbid waters. The 50 dB threshold marks the effective transmission limit. Coastal and turbid waters show rapid SNR loss, while pure and clear waters maintain higher SNRs over longer distances. LD-PS generally performs better, highlighting its efficiency in UOWC systems.

Table 7 presents the link range for LED-PS and LD-PS at an SNR threshold of 50 dB under different turbulence models across water types.

**Table 7:** The achievable link range for LED-PS and LD-PS under different turbulence models across various water types at (SNR = 50 dB).

Water Type	Turbulence Model	Link Range for (LED-PS) in (m) at SNR = 50 dB	Link Range for (LD-PS) in (m) at SNR = 50 dB
Pure Sea	Log-Normal	63.53	73.34
	Generalized Gamma	47.58	56.2
	Weibull	21.81	27.56
Clear Ocean	Log-Normal	38.95	43.91
	Generalized Gamma	30.63	35.19
	Weibull	16.16	19.53
Coastal Ocean	Log-Normal	17.25	18.94
	Generalized Gamma	14.38	15.96
	Weibull	8.83	10.22
Turbid Harbor	Log-Normal	4.96	5.26
	Generalized Gamma	4.27	4.67
	Weibull	3.08	3.38

The following conclusions can be drawn:

Across all water types, the log-normal model offers the longest link range, followed by generalized gamma, with Weibull resulting in the shortest. This indicates log-normal represents less severe turbulence, while Weibull reflects strong fading effects. In turbid harbor water, the link range is severely reduced for all models due to high scattering and absorption. LD-PS consistently outperforms LED-PS due to its higher coherence and directivity, improving signal strength and turbulence resilience. The performance gap is more pronounced in clearer waters. As turbidity increases, both sources experience sharp range reductions, with the most severe attenuation in turbid harbor water.

These findings stress the need to choose appropriate sources and models when designing UOWC systems. LD-PS is preferable for long-range communication, especially in clear waters. The choice of turbulence model is also crucial, with log-normal providing the most optimistic estimates and Weibull offering a conservative benchmark under harsh conditions.

**Table 8:** The required SNR for LED-PS and LD-PS under different turbulence models across various water types at (BER = 10^−5^).

Water Type	Turbulence Model	SNR for (LED-PS) in (dB) at BER = 10^−5^	SNR for (LD-PS) in (dB) at BER = 10^−5^
Pure Sea	Log-Normal	13.96	12.19
	Generalized Gamma	19.61	17.84
	Weibull	31.41	29.65
Clear Ocean	Log-Normal	16.04	14.29
	Generalized Gamma	21.71	19.95
	Weibull	33.51	31.75
Coastal Ocean	Log-Normal	24.60	22.84
	Generalized Gamma	30.25	28.50
	Weibull	42.06	40.30
Turbid Harbor	Log-Normal	76.24	74.48
	Generalized Gamma	81.90	80.13
	Weibull	93.70	91.94

**Fig. 13:**
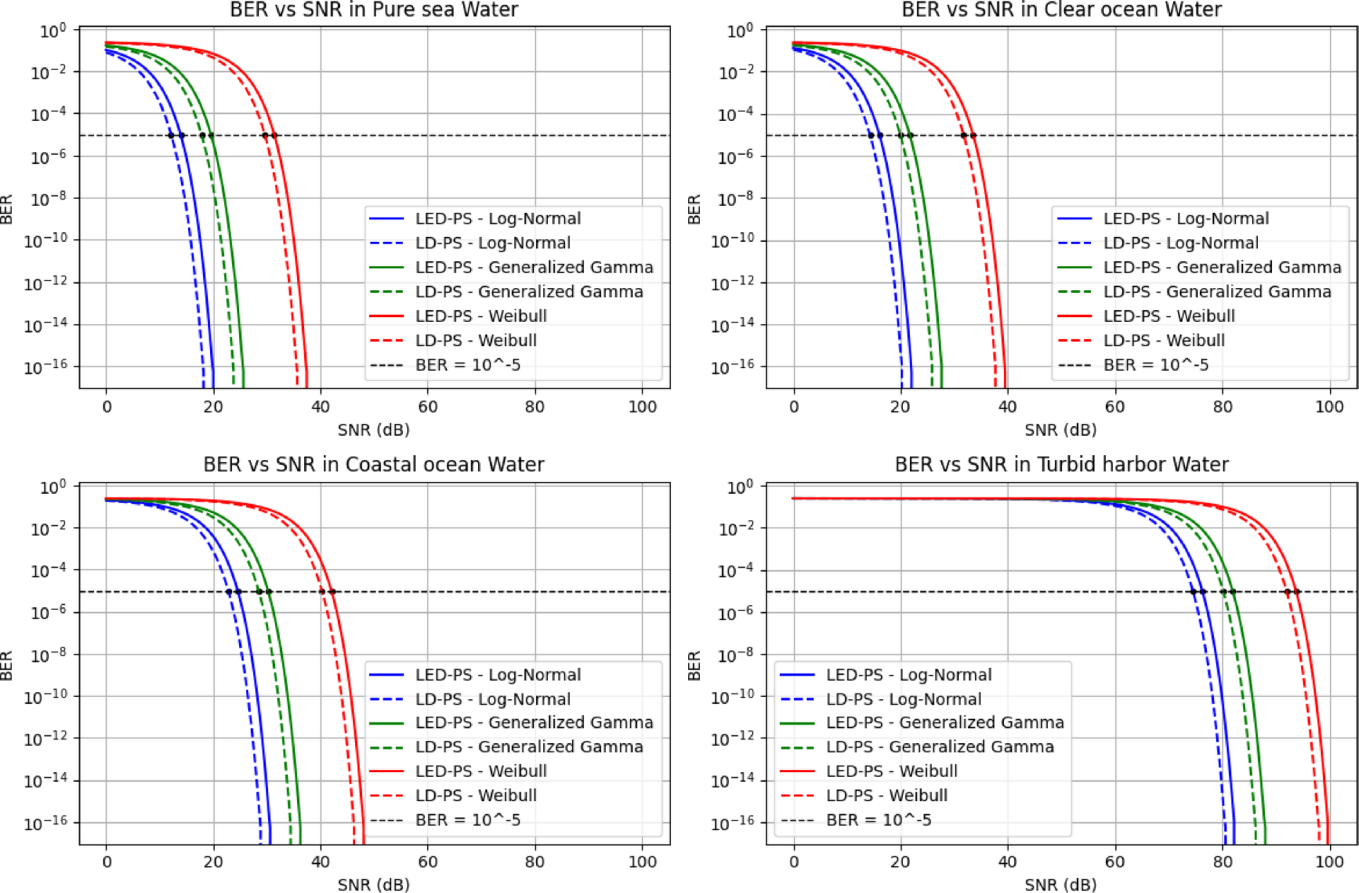
BER versus SNR (dB) for different water types under various turbulence models and photo-sources.

Figure 13 presents BER vs. SNR in different water types for LED-PS and LD-PS under log-normal, generalized gamma, and Weibull turbulence. As turbidity increases, a higher SNR is required to maintain a low BER. Pure and clear waters show better performance, while coastal and turbid waters need higher SNR. In turbid harbor water, BER remains high, indicating severe signal degradation. LD-PS generally outperforms LED-PS, especially under the log-normal model.

Table 8 provides the required SNR to achieve BER = 10^-5^ using LED-PS and LD-PS under different turbulence models and water types.

The conclusions are:

### Effect of water type on required SNR

Required SNR increases as water clarity decreases (Pure Sea < Clear Ocean < Coastal Ocean < Turbid Harbor). For example, in pure seawater under log-normal turbulence, the required SNR is 13.96 dB (LED-PS) and 12.19 dB (LD-PS). In turbid harbor water, it rises to 76.24 dB (LED-PS) and 74.48 dB (LD-PS), demonstrating the need for stronger transmission or advanced signal processing in turbid environments.

### Impact of turbulence models on required SNR

The Weibull model requires the highest SNR, followed by generalized gamma, with log-normal needing the lowest. For instance, in coastal ocean water, log-normal requires 24.60 dB (LED-PS) and 22.84 dB (LD-PS), while Weibull needs 42.06 dB and 40.30 dB, respectively.

### Comparison between LED-PS and LD-PS

LD-PS always requires a lower SNR than LED-PS due to better coherence and directionality, reducing losses and improving detection. The benefit is more noticeable in high-turbidity conditions. In turbid harbor water under Weibull turbulence, LD-PS requires 91.94 dB vs. 93.70 dB for LED-PS.

### Practical implications

This study highlights the importance of selecting suitable optical sources and turbulence models in the design of UOWC systems. In relatively clear waters (pure Sea and clear Ocean), both LED-PS and LD-PS achieve acceptable SNR levels, supporting their use in practical deployments. In contrast, highly turbid environments (coastal ocean and turbid harbor) demand significantly higher SNR, indicating a need for advanced techniques such as adaptive modulation, enhanced signal processing, or hybrid communication methods to sustain performance. Among the evaluated turbulence models, the Weibull model consistently imposes the highest SNR demands, making it a critical consideration for worst-case design scenarios. Across all conditions, LD-PS outperforms LED-PS, reinforcing its suitability for challenging underwater environments.

These findings provide a foundation for optimizing UOWC system design based on environmental conditions. Future research could focus on adaptive power control, error correction, and machine learning-driven approaches to mitigate performance degradation in turbid waters.

*Finally*, Table 9 directly contrasts the outcomes of our proposed model with selected recent studies. This comparison focuses on key performance metrics such as:

- Achievable link range under various water types.
- Required SNR at a target BER.
- Use of turbulence models and photo-source types.

**Table 9:** Comparison of our proposed UOWC simulation model with recent works.

Study	Water Types	Turbulence Models	Transmitter	Max Link Range (m)	BER Target	Required SNR (dB)	Notable Features
^18^	Clear, Coastal, Harbor	Log-normal	517 nm Laser	6–35	10 – 10⁻^12^	Not stated	Monte Carlo-based BER evaluation
^21^	Coastal, Turbid Harbor	Log-Normal, Gamma, Weibull	532 nm Laser and LED-PS	25 (laser), 6 (LED)	10^⁻6^	~ 25	Turbulence fading analysis
^28^	Pure Seawater, Ocean, Coastal	Normal, log-normal, logistic, Weibull	450 nm Laser	50	10^⁻6^	Not stated	Power penalty analysis
^32^	Tap Water, Artificial Seawater	Not specified	450 nm Blue Laser	6	10^⁻8^	N/A	Real experiment, 1.25 Gbps data rate
The current work	Pure Sea, Clear, Coastal, Turbid Harbor	Log-Normal, Gen. Gamma, Weibull	520 nm LED-PS, LD-PS	Up to 73.34	10^⁻5^	12.19–91.94	Comprehensive turbulence + noise modeling

While this study does not employ a neural network architecture, we implement an *ablation-style analysis* by systematically evaluating the contribution of individual physical components of the system. This includes isolating the effects of different turbulence models, water types, photo-source configurations (LED-PS vs. LD-PS), and noise sources on key performance metrics such as BER, SNR, and communication range. These controlled evaluations, reflected in Tables 5, 6, 7 and 8; Figs. 10, 11, 12 and 13, validate the sensitivity and importance of each parameter in determining UOWC system performance.

### Computational cost and complexity analysis

The computational complexity of the proposed simulation framework is primarily driven by the number of iterations over environmental parameters and stochastic realizations of fading. For each simulation batch (i.e., fixed source, water type, and turbulence model), the runtime scales linearly with the number of distance points (N) and Monte Carlo samples (M), leading to an overall complexity of O (N × M).

Compared to basic exponential attenuation models (O(N)) or log-normal-only fading approximations, our approach remains computationally lightweight while providing deeper accuracy by integrating multiple turbulence models and noise sources. Table 10 provides a comparison of the computational characteristics and fidelity of our framework relative to traditional methods.

**Table 10:** Complexity and capability comparison.

Method	Channel Effects Modeled	Noise Included	Turbulence Modeled	Complexity	Comment
Simple exponential decay model	Attenuation only	X	X	O(N), low	Fast but unrealistic
Log-normal only	Attenuation + fading	Limited	✓ (only 1 model)	O(N×M), low	Used in earlier UOWC works
ML-based channel estimation (e.g., ANN)	Varies	Implicit	Indirectly learned	High (training time)	Not interpretable, requires dataset
**The current work**	**Absorption**,** scattering**,** noise (thermal**,** dark**,** RIN)**,** turbulence (3 models)**	**✓**	**✓ (3 statistical models)**	**O(N×M)**,** moderate**	**Comprehensive but lightweight**

All simulations were conducted using MATLAB R2023a and Python 3.10 on a standard Intel Core i7 machine with 16 GB RAM. No GPU acceleration was used, as the simulations are based on closed-form equations and lightweight Monte Carlo sampling.

## Conclusion and future work

This study presents a comprehensive analysis of UOWC channels for IoUT applications, focusing on the combined effects of water types, turbulence models, and transmitter configurations. Through detailed modeling and simulation, the performance of LED-PS and LD-PS, operating at a 520 nm wavelength, was evaluated across diverse aquatic environments, including pure seawater, clear coastal waters, and turbid harbor waters. By integrating three statistical turbulence models—log-normal, generalized gamma, and Weibull distributions—the study offers a realistic assessment of underwater optical link performance under varying channel impairments. The findings highlight the critical impact of absorption, scattering, and turbulence-induced fading on link reliability, underscoring the need for adaptive and environment-aware communication strategies in IoUT networks. The key outcomes of this study can be summarized as follows:

- LD-PS consistently outperforms LED-PS across all water types and turbulence models, particularly in terms of received power and achievable communication distance.
- LD-PS achieves a maximum communication distance of 73.34 m in pure seawater using the log-normal model—over 15.45% longer than that of LED-PS under the same conditions.
- Under a BER threshold of 10^-5^, LD-PS requires a minimum SNR of only 12.19 dB in pure sea water under the log-normal model, compared to a maximum of 93.70 dB required by LED-PS in turbid harbor water under the Weibull model—demonstrating LD-PS’s superior energy efficiency, especially in clearer environments.
- The generalized gamma turbulence model yields more conservative BER predictions than the log-normal model but is less severe than the Weibull model. For worst-case performance estimation—particularly in turbid harbor waters—the Weibull model is more appropriate due to its stronger representation of severe turbulence effects.
- Among turbulence models, the log-normal distribution generally provides the longest communication range, while the Weibull model reflects the most severe fading.
- As water turbidity increases, communication range deteriorates significantly, with the sharpest performance decline observed in turbid harbor conditions.
- LED-PS remains useful in highly dynamic or misaligned environments due to its wider beam, despite its shorter range and higher SNR requirements.

Future research can extend this work by exploring adaptive modulation techniques and machine learning-based predictive models to dynamically optimize system performance in varying underwater environments. Experimental validation using real-world underwater testbeds is essential to further assess practical deployment challenges and validate the simulation results. In addition, the integration of hybrid communication architectures—combining optical, acoustic, and RF technologies—can enhance the overall robustness, range, and redundancy of IoUT networks. Investigating advanced error correction methods and power-efficient transmission strategies will also be critical for enabling sustainable long-term operations, particularly for autonomous underwater vehicles (AUVs) and remote sensor nodes. These directions will contribute to the development of resilient, high-capacity IoUT systems capable of supporting reliable and efficient real-time data transmission across a broad spectrum of underwater applications, including environmental monitoring, underwater exploration, and maritime surveillance.

## Data Availability

“The datasets used and/or analysed during the current study available from the corresponding author on reasonable request.”
